# *Drosophila* Genes That Affect Meiosis Duration Are among the Meiosis Related Genes That Are More Often Found Duplicated

**DOI:** 10.1371/journal.pone.0017512

**Published:** 2011-03-10

**Authors:** Micael Reis, Sofia Sousa-Guimarães, Cristina P. Vieira, Cláudio E. Sunkel, Jorge Vieira

**Affiliations:** Instituto de Biologia Celular e Molecular (IBMC), University of Porto, Porto, Portugal; University of Wyoming, United States of America

## Abstract

Using a phylogenetic approach, the examination of 33 meiosis/meiosis-related genes in 12 *Drosophila* species, revealed nine independent gene duplications, involving the genes *cav*, *mre11*, *meiS332*, *polo* and *mtrm*. Evidence is provided that at least eight out of the nine gene duplicates are functional. Therefore, the rate at which *Drosophila* meiosis/meiosis-related genes are duplicated and retained is estimated to be 0.0012 per gene per million years, a value that is similar to the average for all *Drosophila* genes. It should be noted that by using a phylogenetic approach the confounding effect of concerted evolution, that is known to lead to overestimation of the duplication and retention rate, is avoided. This is an important issue, since even in our moderate size sample, evidence for long-term concerted evolution (lasting for more than 30 million years) was found for the *meiS332* gene pair in species of the *Drosophila* subgenus. Most striking, in contrast to theoretical expectations, is the finding that genes that encode proteins that must follow a close stoichiometric balance, such as *polo*, *mtrm* and *meiS332* have been found duplicated. The duplicated genes may be examples of gene neofunctionalization. It is speculated that meiosis duration may be a trait that is under selection in *Drosophila* and that it has different optimal values in different species.

## Introduction

Gene duplication followed by the fixation of a mutation providing a different function, is one of the major sources to create genetic novelty [1]. The rate at which eukaryotic duplicated genes are retained, i.e., go to fixation, has been originally estimated to be 0.01 per gene per million years [2]. This value was obtained under the assumption that the age of the duplication events can be estimated by looking at within species synonymous divergence rates between pairs of paralogous genes. Nevertheless, this estimate is inflated due to concerted evolution [1], [3]. Concerted evolution arises due to frequent gene conversion between paralogous genes. This process leads to a severe reduction in the divergence rate between paralogous genes from the same species but not when comparing different species (see for instance [4]). Using species of the *D. melanogaster* subgroup, and taking into account the effect of concerted evolution, i.e, using a phylogenetic approach, Osada and Innan [1], estimated the rate of duplication to be 0.001 per gene per million years, an order of magnitude below the original estimate.

Not all gene duplicates are predicted to be equally retained. For instance, duplication of genes, that encode for proteins that are part of a complex, are likely deleterious [5], [6], [7]. Moreover, theory suggests that, duplication of genes that encode for proteins involved in regulatory networks are rarely retained, since they likely disrupt network dynamics and consequently the expression pattern of many genes [8]. Duplications of genes encoding for proteins involved in signaling networks are also expected to be rarely retained [8]. Gene duplicates that encode for proteins that participate in many reactions are, as well, less likely to be retained than genes that encode proteins that participate in a single reaction [9]. Duplicates of genes that encode for activators are also expected to be more frequently retained than genes that encode for receptors [7]. In *Drosophila*, developmental constraint, for instance, does appear to reduce gene duplicability, but the effect is moderate [10].

How the gene duplicates came to be also influences gene duplicate retention. For instance, in *Arabidopsis*, when large-scale duplication events are involved, genes that encode transcription factors, proteins with kinase activity, proteins that are involved in protein binding and modification, or in signal transduction pathways are retained at high rates, but the same categories are retained at low rates when small-scale duplications are involved [11]. As discussed by Maere *et al.* [11] large scale duplication events may not disrupt stoichiometric balances, while small-scale duplication events likely do. In *Drosophila*, however, most duplication events seem to involve less than four genes, and for the vast majority of blocks, the length of the region between the original and the duplicated block is less than 5 Kb [1]. No large scale duplications have ever been described in *Drosophila*.

Many meiotic pathways are highly conserved across distantly related sexually reproducing eukaryotes (for a review, see [12]). Such conservation could mean that meiotic pathways tolerate little change. Moreover, in *Arabidopsis*, duplicates of genes involved in DNA repair, DNA replication, DNA recombination, and cell-cycle genes are generally little retained [11]. This is not surprising since meiosis-related genes are known to participate in multiple pathways, be involved in protein complexes, and, when disrupted, affect multiple aspects of meiosis (see Table S1). Nevertheless, the time it takes to complete meiosis is known to be very variable, even among species without developmental holds. Environmental factors (temperature for instance), nuclear DNA content and genotype are among the most important factors affecting meiosis duration [13]. In *Drosophila*, nuclear DNA content is known to vary significantly (the C-values vary between 0.12 and 0.39; http://www.genomesize.com), but meiosis duration has been recorded so far only in *D. melanogaster* [13]. Unusual prophase structures such as fibrillar structures apparently coupled to the nucleolus, and multiple nucleoli are also observed in species of the *virilis* group [14]. These observations suggest that, even within a single genus, such as *Drosophila,* meiosis features are after all variable. Whether *Drosophila* meiosis-related neomorphs (meiosis-related genes with new functions) have evolved is also unknown. This is important, in order to infer the tolerated degree of change of an ancient machine such as the meiotic one.

Recently, Anderson *et al.* [15] studied 33 genes involved in meiosis or meiosis-related tasks, such as, chromosome segregation, achiasmate segregation, crossover regulation, double-strand-break formation, heterochromatin binding, recombination and/or repair, sister-chromatid cohesion, spindle assembly, and telomere maintenance. That study revealed that, in *Drosophila*, variability patterns compatible with adaptive protein divergence and polymorphism can be found at four meiosis (*Klp3A*, *Ku80*, *mtrm*, and *ord*) and two telomere maintenance genes (*mre11* and *rad50*). Nevertheless, as argued by Anderson *et al.* [15], the observed patterns can also be explained as a consequence of the fixation/persistence in *Drosophila* populations, of meiotic drive elements (elements that in females influence the preferential sorting of a chromosome to the pronucleus, and thus to the ovule; [15]). If meiotic drive elements are common (about 18% of the meiotic genes surveyed by Anderson *et al.* [15] could show evidence for meiotic drive elements), then such elements could conceivably also increase the probability of fixation, and thus the retention of meiosis gene duplicates. It should be noted, however, that the extent to which the observed within and between species amino acid variation at meiosis genes is adaptive is unknown.

In this work, in order to avoid the confounding effect of concerted evolution (see above), a phylogenetic approach is used for the estimation of the rate at which meiosis-related genes are duplicated and retained. A segmental duplication may lead to the simultaneous duplication of many neighboring genes. When segmental duplications are not taken into account, the gene duplication rate is overestimated. Therefore, in this work, the time of origin, as well as the lineage where the gene duplication occurred, is also taken into account, when inferring the number of independent gene duplication events. Due to the methodological approach used, only gene duplications that occurred after the separation of the *Drosophila* and *Sophophora* sub-genera are counted. Recent gene duplicates are expected to be found in tandem, unless they are the result of a segmental duplication, or retrotransposition is involved. Nevertheless, the separation of the two *Drosophila* subgenera occurred about 40 million years ago [16]. Therefore, a fraction of the inferred gene duplications may be old. Because gene order can be shuffled due to inversions and translocations, those duplications are no longer expected to be in tandem. Moreover, we infer whether the gene duplicates are functional, since such genes are potential meiosis-related neomorphs. We speculate on whether variation in meiosis gene copy numbers, as well as the appearance of putative neomorphs, can account for the variability in *Drosophila* meiosis features, although these findings must be corroborated by detailed functional studies.

## Materials and Methods

### Strains

*D. virilis* 1051.49 (Chaco, Argentina); *D. persimilis* 14011-0111.48 (California, USA); *D. willistoni* 14030-0811.16 (Rocha, Uruguay) and *D. mojavensis* 15081-1352.00 (California, USA) were used to address the expression profile of the different genes found to be duplicated and their respective duplicates. Furthermore, in order to determine the age of the *mtrm* gene duplication (*mtrm-dup*) the following species from the *virilis* group of *Drosophila* were used: *D. novamexicana* 15010-1031.00 (Colorado, USA), *D. lummei* 200 (Russia), *D. littoralis* BP41 (Bragança, Portugal), *D. kanekoi* 15010-1061.00 (Sapporo, Japan), *D. ezoana* E20 (Kemi, Finland), *D. montana* Mo1 (Kemi, Finland), *D. flavomontana* 15010-0981-00 (Idaho, USA), *D. lacicola* 15010-0991-00 (New York, USA), *D. borealis* 15010-0961-00 (Minnesota, USA) and *D. borealis* 15010-0961-03 (Idaho, USA). To test the hypothesis of preferential transmission of chromosomes having one of the variants at *mtrm-dup* gene, the following strains were used: *D. a. americana* NN97.4, NN97.8 (Nebraska, USA), W11, W23 (Lake Wappapelo, USA) and *D. a. texana* W29 (Lake Wappapelo, USA), LP97.7 (Louisiana, USA), ML97.5; ML97.4.2 (Louisiana, USA).

### Genomic DNA extraction

Genomic DNA from single males was extracted using the QIAamp DNA Mini Kit from QIAGEN (Izasa Portugal, Lda.) according to the manufacturer's instructions.

### PCR amplification

Specific primers were developed for each of the genes found to be duplicated and their respective duplicates (Table S2). To test the hypothesis of preferential transmission of chromosomes having one of the variants at *mtrm-dup* this gene was amplified in the species from the *virilis* group of *Drosophila* using primers 543F690 and 543R43 as described in Vieira *et al.* [17]. Standard amplification conditions were 35 cycles of denaturation at 94°C for 30s, primer annealing according to Table S2, for 45s, and primer extension at 72°C for 3min.

### RT-PCR

Ovaries and testes were dissected from *D. virilis* (1051.49), *D. willistoni* (14030-0811.16), *D. mojavensis* (15081-1350.00) and *D. persimilis* (14011-0111.48). Total RNA was isolated from the dissected tissues using TRIzol Reagent (Invitrogen) according to the manufacturer's instructions and treated with *DNase I* (*RNase*-Free) (Ambion). cDNA was synthesized by reverse transcription with SuperScript III First-Strand Synthesis SuperMix for qRT-PCR (Invitrogen). cDNAs were amplified using the PCR conditions described above and the specific primers shown on Table S2. Specific primers were also used for the endogenous *ribosomal protein L32* (*RpL32*) as a control for cDNA quality. No-template controls and reactions with RNA that was not reverse transcribed were performed in order to confirm the absence of genomic DNA contamination. Moreover, when possible, primers were designed in order to encompass a region of the gene with one intron. Therefore, the cDNA amplification product is expected to have a shorter size than the amplification product from genomic DNA. The results were analyzed by agarose gel electrophoresis. It should be noted that expression levels of different genes should not be compared since, for instance, amplification product sizes are different, and primer features (such as GC content, or melting temperatures) are different. Direct sequencing was performed using all the PCR products obtained from cDNA amplification as template to confirm the specificity of the primers developed for all the genes found to be duplicated and their respective duplicates. Moreover, for a given gene and its duplicates, when using cDNA, most PCR amplification products have different sizes.

### Sequencing

The amplification products obtained for the species from the *virilis* group of *Drosophila* using primers 543F690 and 543R43 [17] were cloned using the TOPO-TA Cloning Kit for Sequencing from Invitrogen (Barcelona, Spain). Positive colonies were picked randomly, grown in 5mL of LB with Ampicillin, and plasmids were extracted using the QIAprep Spin Miniprep Kit from QIAGEN (Izasa, Portugal, Lda.). Four colonies were sequenced in order to correct for possible nucleotide missincorporations that may have occurred during the PCR reaction. Sequencing was performed using ABI PRISM Big Dye cycle-sequencing kit version 1.1 (Perkin Elmer, CA, USA) and the primers for the M13 forward and reverse priming sites of the pCR2.1 vector. Sequencing runs were performed by STABVIDA (Lisbon, Portugal).

### Restriction enzyme typing of a common polymorphism on the *mtrm-dup* gene

To test the hypothesis of preferential transmission of chromosomes having one of the amino acid variants at *mtrm-dup* gene [17] a total of 32 crosses were established corresponding to all possible combinations between *D. a. americana* and *D. a. texana* strains (F0) in both directions. After emergence of new born individuals brother-sister mating was performed (F1). All the females were heterozygous for the *mtrm-dup* amino acid variants. In the next generation (F2) 10 males from each of the F1 crosses established were selected in a total of 320 individuals. The genomic DNA from these individuals was extracted and they were genotyped for the presence of the amino acid variant on *mtrm-dup* associated with the *X*/*4* fusion, using the restriction enzyme *Bst*BI and the PCR amplification products obtained with primers 543F69 and 543R43 (see [17]).

### Datasets, sequence alignment and phylogenetic analyses

The *D. melanogaster* coding sequences of the 33 meiosis-related genes listed in [15], was retrieved from FlyBase (http://flybase.org/). In order to retrieve sequences from non-*melanogaster Drosophila* species, the tblastn option with standard parameters, as implemented in FlyBase, was used. The *D. melanogaster* coding sequences were used as a query. Coding sequences with an associated expected value less than 0.05 were retrieved. When gene sequences were non-annotated, a tentative manual annotation of the putative coding region was performed. For every gene dataset, translated amino acid sequences were aligned using CLUSTALW, as implemented in DAMBE [18]. The resulting amino acid alignment was used as a guide to obtain the corresponding nucleotide alignment. Bayesian trees were obtained using MrBayes [19], and nucleotide sequences, under the GTR model of sequence evolution, thus allowing for among-site rate variation and a proportion of invariable sites. Third codon positions are allowed to have a gamma distribution shape parameter that is different from that of first and second codon positions. Two simultaneous and completely independent analyzes, starting from random trees, were run for 500,000 generations (each with one cold and three heated chains). Samples were taken every 100th generation. The first 1250 samples were discarded (burn-in). The final datasets (accession numbers for the nucleotide sequences used can be found in Table S3) were obtained after inspecting the results of the phylogenetic analyses. Because of the methodology used, only gene duplications that occurred after the separation of the *Drosophila* and *Sophophora* sub-genera are counted. Since our goal was to estimate the rate of duplication of meiosis-related genes, whether the duplicated genes were created as a result of the duplication of a segment of the genome (segmental duplications) or as the result of the duplication of a single gene was not assessed. Nevertheless, given the inferred time of origin and the lineage where gene duplications are inferred to have occurred, the detected gene duplications must be the result of independent duplication events (see Results section).

### Divergence estimates

Per site non-synonymous (*K_a_*) and synonymous (*K_s_*) rates were estimated using DNasp [20]. Values are Jukes-Cantor corrected for multiple hits.

### Tajima's relative rate tests

In order to infer whether duplicated genes evolve at the same rate as the gene that was duplicated, Tajima's relative rate tests were performed, as implemented in the MEGA software [21], using all codon positions, or third codon positions (those most likely to be neutral) only. For this test, two ingroup and one outgroup sequences are used. Under the molecular clock hypothesis, irrespective of the substitution model and whether or not the substitution rate varies with the site, the number of mutations inferred for the two ingroup branches should be similar. If this hypothesis is rejected, then the molecular clock hypothesis can be rejected for this set of sequences. When the two ingroup sequences have different amino acid constraints but are subject to a similar mutation rate, statistically significant differences are expected when using all codon positions but not when using third codon positions [22].

## Results

### The vast majority (85%) of the genes involved in meiosis related tasks are not duplicated

**Figure 1:**
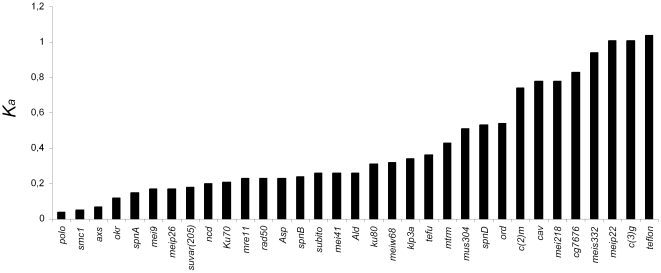
Jukes-Cantor corrected per site rate of non-synonymous substitutions between *D. melanogaster* and *D. virilis* for 33 meiosis genes. For *CG7676* gene this value has been extrapolated under the assumption of a molecular clock and that *D. melanogaster* and *D. virilis* have been diverging for about 40 million years while *D. melanogaster* and *D. yakuba* have been diverging for about 10 million years.

**Figure 2:**
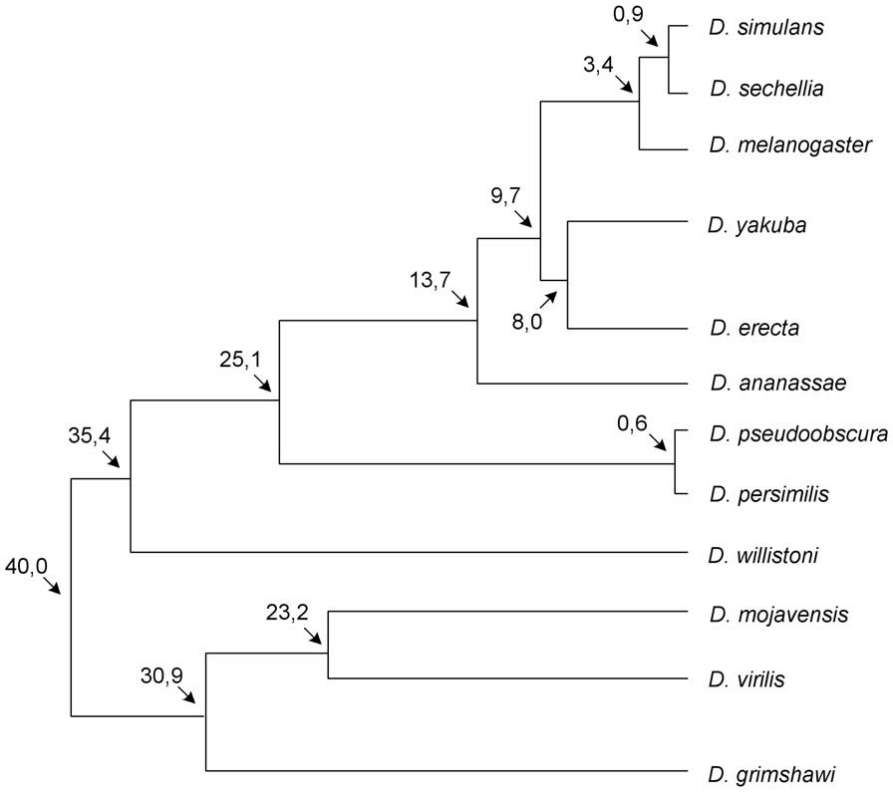
Relationship of the *Drosophila* species studied. Adapted from http://flybase.org. Numbers are estimated divergence times in million years.

Of the 33 meiosis-related genes studied (those listed in [15]), 31 could be found in the 12 publicly available *Drosophila* genomes (http://flybase.org/) although a non-negligible fraction is non-annotated or likely miss-annotated (Table S4). The *c(3)G* gene could not be found in *D. ananassae*. Nevertheless, it is found in all other species examined and thus, it is likely that the *D. ananassae* genomic region encompassing gene *c(3)G* has not been sequenced. Gene *CG7676* (also known as *cona*; http://flybase.org/) could not be found in *D.ananassae*, *D. willistoni*, *D. mojavensis*, *D. virilis* and *D. grimshawi*. Therefore, the latter gene is never found in species of the *Drosophila* subgenus. In Fig. 1, the per site non-synonymous rate of evolution between *D. melanogaster* and *D. virilis* is shown for the 33 meiosis-related genes. For *CG7676* gene this value has been extrapolated under the assumption of a molecular clock and that *D. melanogaster* and *D. virilis* have been diverging for about 40 million years while *D. melanogaster* and *D. yakuba* have been diverging for about 10 million years (see Fig. 2). *CG7676* is not evolving faster than other meiosis-related genes that have a clearly recognizable orthologous copy in *D. virilis* (Fig. 1). Therefore, we should have been able to detect the *CG7676* orthologous copy in species of the subgenus *Drosophila*. Given these observations it seems likely that gene *CG7676* does not have an orthologous copy in the subgenus *Drosophila*, an unexpected observation for a gene involved in a tightly regulated process. This gene has been described as being required for the stable ‘zippering’ of transverse filaments to form the central region of the *Drosophila* synaptonemal complex [23].

For 26 (*ald, asp, Axs, c(2)M, c(3)G, Su(var)205, Klp3A, Ku70, Ku80, mei-218, mei-41, mei-P22, mei-P26, mei-9, mus304, ncd, okr, ord, rad50, smc1, spn-A, spn-B, spn-D, subito, teflon* and *tefu*) out of the 33 genes analyzed, there is a single copy in the 12 *Drosophila* genomes and thus there is no evidence for gene duplications. For two genes (*MeiW68* and *CG7676*) two copies could be found in *D. sechellia* and *D. yakuba*, respectively. It is, however, likely that these are artifacts of the genome assembly process. Indeed, the two *MeiW68* gene copies are identical at the nucleotide level and the duplicated copy is located on a small scaffold that has not been anchored to any chromosome. The two proteins encoded by gene *CG7676* are 194 and 190 amino acids long. Besides the indel, there is a single nucleotide difference between the two coding sequences. It should be noted that the shorter putatively duplicated gene is located on a small scaffold that has not been anchored to any chromosome. Therefore, we conclude that there is no solid evidence for *MeiW68* and *CG7676* gene duplications. Genus-wide, 85% of the meiosis-related genes do not have duplicates. However, nine independent gene duplications involving the genes *cav*, *mre11*, *meiS332*, *polo* and *mtrm* were found. The 12 *Drosophila* species here analyzed imply about 230 million years of independent evolution (Fig. 2). Therefore, *Drosophila* meiosis-related genes are duplicated at a rate of 0.0012 per gene per million years. This rate is similar to that estimated for the whole *Drosophila* genome [1].

In what follows, for each gene showing duplicates, their evolutionary history, as well as evidence that the gene duplicate(s) are functional is presented.

### Three independent *cav* gene duplications

**Figure 3:**
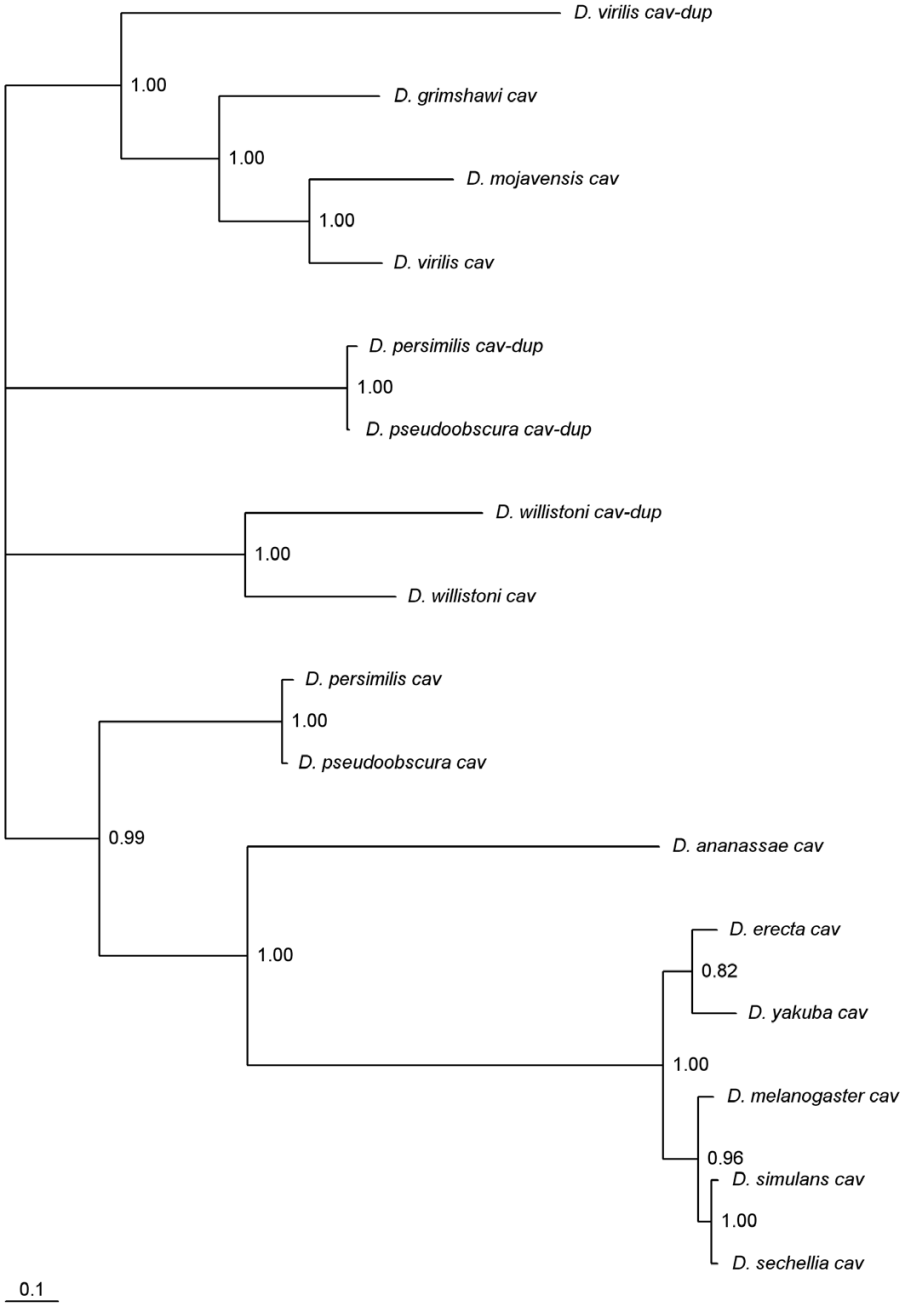
Bayesian phylogram of *Drosophila cav*-like genes. Numbers are posterior credibility values.

**Figure 4:**
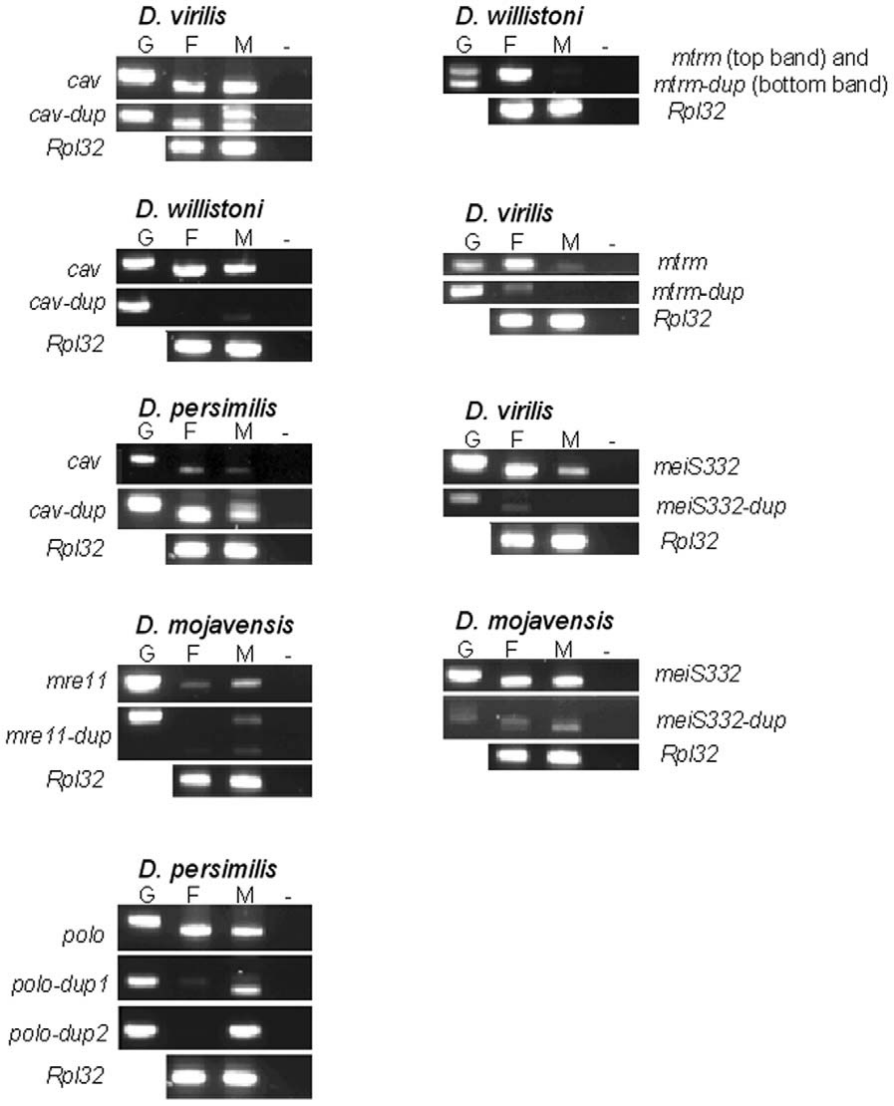
Expression patterns of genes found to be duplicated. G – genomic DNA; F – female gonads; M – male gonads. The cDNA of duplicated genes were sequenced in order to assure amplification specificity. In males, for genes *D. virilis cav-dup*, *D. persimilis cav-dup*, *D. mojavensis mre-11-dup* and *D. persimilis polo-dup1* a band with the size expected for an amplification from genomic DNA is observed. In order to rule out the possibility of contamination with genomic DNA, the PCR reactions were performed three times independently starting from different cDNA aliquots and identical results were obtained. The observation that when using the same aliquot, the duplicated gene shows two bands but the genes *D. virilis cav*, *D. persimilis cav*, *D. mojavensis mre-11* and *D. persimilis polo* show only one band of the expected size also shows that there is no contamination with genomic DNA.

cav is a DNA-binding protein that is a component of the multiprotein *Drosophila* origin recognition complex [24]. Phylogenetic analyses revealed three independent *cav* gene duplications (Fig. 3). There is always a *cav* gene on Muller's element E, thus it seems reasonable to assume that this is the location of the ancestral *cav* gene. In the four species showing two *cav* copies, the duplicated gene is on three different Muller's elements, namely Muller's element A (*D. virilis*), element B (*D. willistoni*) or element E (*D. persimilis, D. pseudoobscura*). This finding is compatible with a scenario invoking three independent duplications, as suggested by the phylogenetic analyses. All *cav* gene duplicates have introns (Table S4), thus retrotransposition seems an unlikely explanation for the observed change in gene location. It should be noted that the phylogenetic tree presented in Fig. 3 implies that the *cav* gene duplication on Muller's element A predates the separation of the *D. grimshawi* and *D. virilis*/*D. mojavensis* lineages, but a duplicated copy cannot be found in either *D. grimshawi* or *D. mojavensis*. Indeed, this *cav* gene duplication is estimated to be as old as the split between the *Sophophora* and *Drosophila* subgenera, and thus about 40 million years old, under the assumption of a molecular clock for synonymous mutations (data not shown). It should be noted that these two *cav* genes are subjected to similar mutation rates but different amino acid constraints (Table 1). The accelerated rate of non-synonymous evolution of the *D. virilis cav-dup* gene (*GJ17001*) could suggest that it is a pseudogene. Nevertheless, this gene is expressed in both males and females (Fig. 4).

**Table 1:** Tajima's relative rate tests using all coding positions or third codon positions only.

Gene	Ingroup species	Outgroup	All positions	Third positions only
*cav*	*D. virilis*	*D. melanogaster*	P<0.001	P>0.05
*cav*	*D. willistoni*	*D. melanogaster*	P>0.05	P>0.05
*cav*	*D. persimilis*	*D. melanogaster*	P>0.05	P>0.05
*cav*	*D. pseudoobscura*	*D. melanogaster*	P>0.05	P>0.05
*mre11*	*D. mojavensis*	*D. grimshawi*	P<0.001	P>0.05
*polo*	*D. persimilis (polo-dup1)*	*D. melanogaster*	P<0.001	P>0.05
*polo*	*D. pseudoobscura (polo-dup1)*	*D. melanogaster*	P<0.001	P>0.05
*polo*	*D. persimilis (polo-dup2)*	*D. melanogaster*	P<0.001	P>0.05
*polo*	*D. pseudoobscura (polo-dup2)*	*D. melanogaster*	P<0.001	P>0.05
*mtrm*	*D. willistoni*	*D. melanogaster*	P>0.05	P>0.05
*mtrm*	*D. virilis*	*D. melanogaster*	P>0.05	P>0.05

There are two *cav* genes in *D. willistoni* that are under similar amino acid constraint, and thus evolving at the same rate (Table 1). This *cav* gene duplication is estimated to be 10 million years old, under the assumption of a molecular clock for synonymous mutations (data not shown). There is no evidence that *cav-dup* is evolving faster than *cav* (Table 1). The duplicated gene seems to be weakly expressed and in males only (Fig. 4). There is thus no evidence that it is a pseudogene.

Two *cav* genes were also found in the two closely related species *D. persimilis* and *D. pseudoobscura*. This *cav* gene duplication is estimated to be 14 million years old, under the assumption of a molecular clock for synonymous mutations (data not shown). There is no evidence that *cav-dup* is evolving faster than *cav* (Table 1). The duplicated gene is expressed in both males and females (Fig. 4).

### A recent *mre11* gene duplication in the *D. mojavensis* lineage

**Figure 5:**
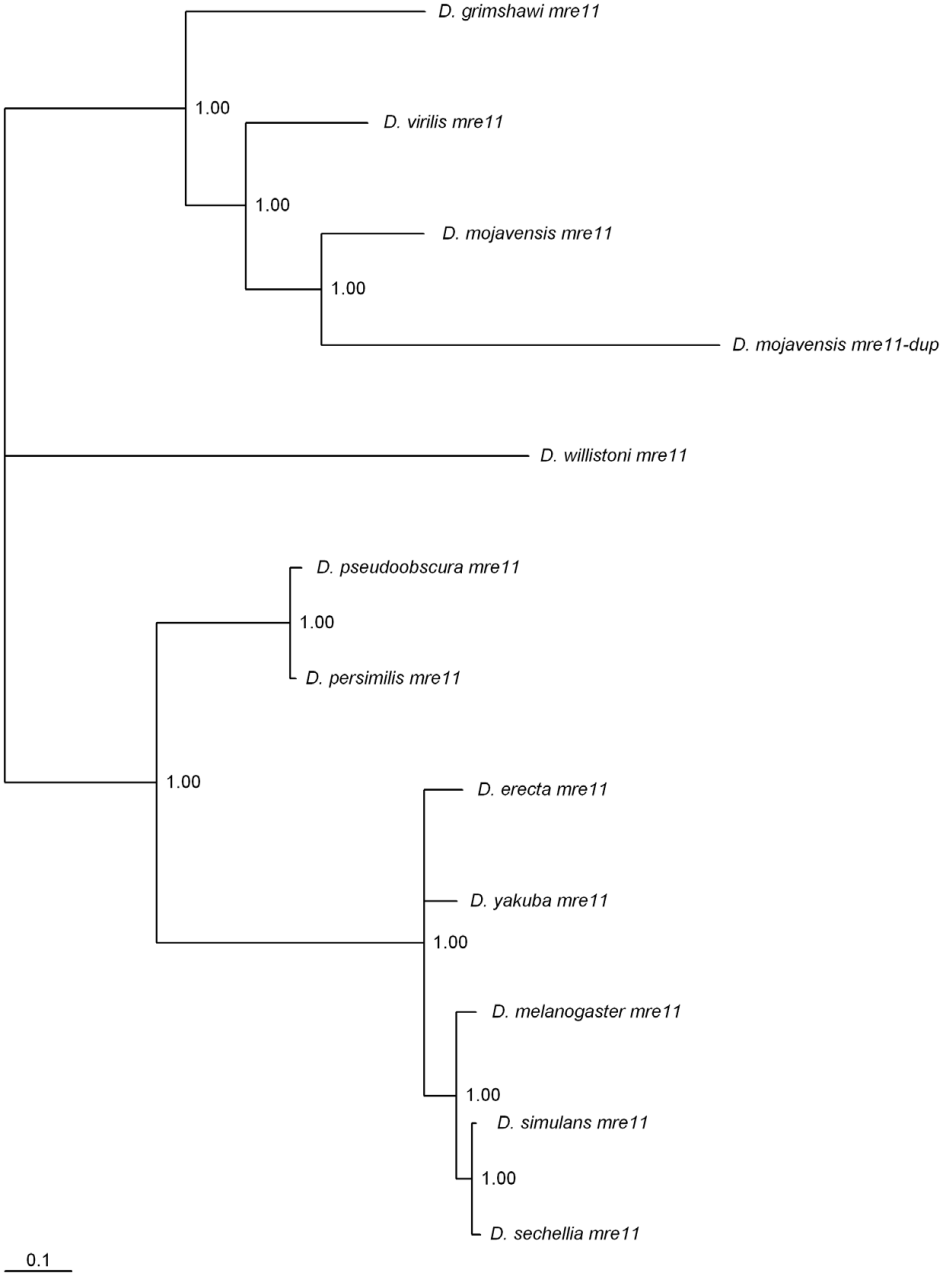
Bayesian phylogram of *Drosophila mre11*-like genes. Numbers are posterior credibility values.

Two *mre11* copies (both on Muller's element B) are found in *D. mojavensis* (Fig. 5). The protein encoded by this gene is involved in telomere maintenance [25]. The *mre11* gene duplication is estimated to be about 15 million years old, under the assumption of a molecular clock for synonymous mutations (data not shown). This duplication occurred in the *D. mojavensis* lineage after the separation from the sister group *D. virilis* lineage. It should be noted that the two *D. mojavensis mre11* genes are subjected to similar mutation rates but different amino acid constraints (Table 1). The accelerated rate of amino acid evolution of the *D. mojavensis mre11-dup* gene (*GI20694*) could suggest that it is a pseudogene. Nevertheless, the *mre11-dup* gene is expressed. *mre11-dup* expression levels are higher in males than in females, a pattern also observed for the *mre11* gene (Fig. 4).

### Two *polo* gene duplications in the *obscura* group

**Figure 6:**
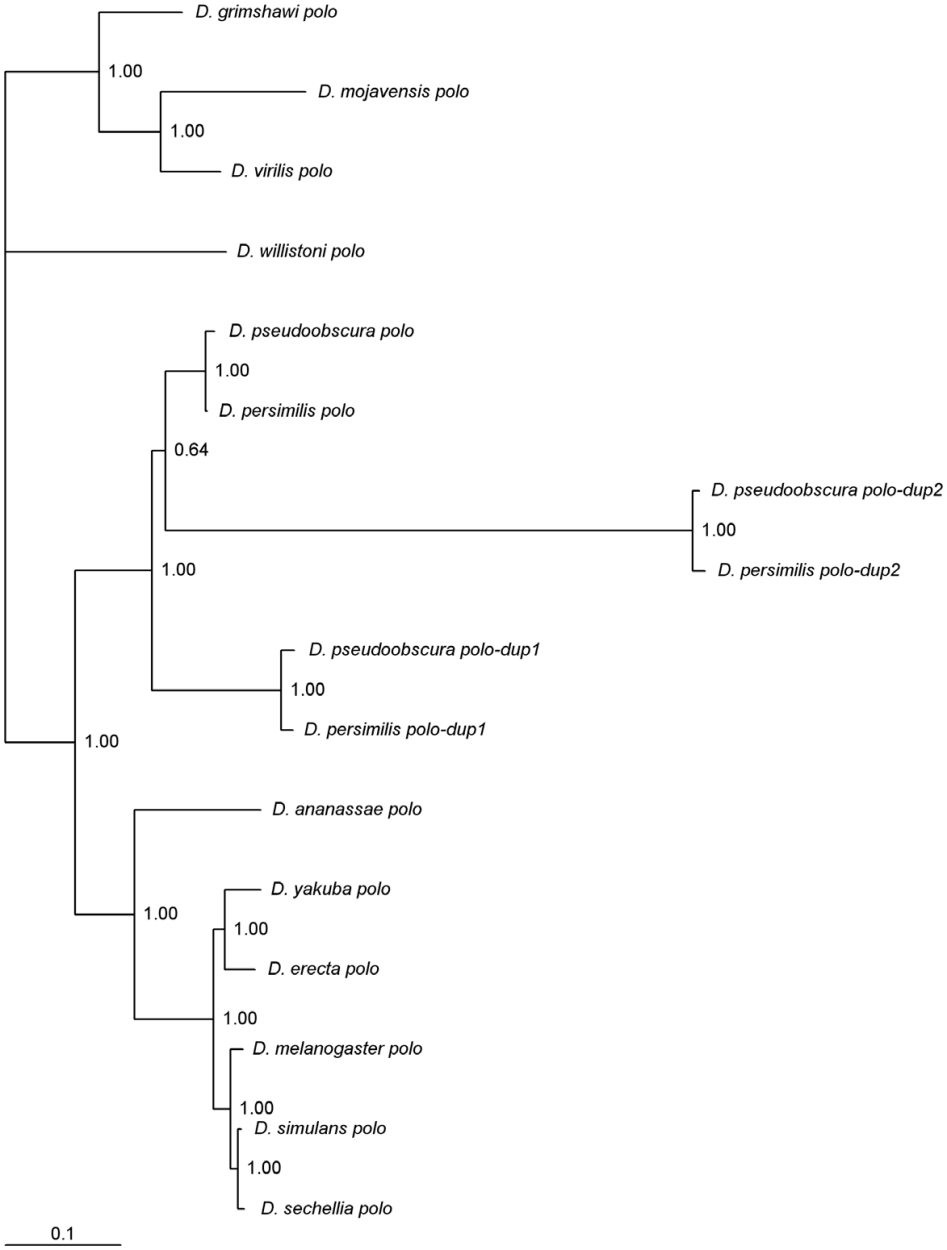
Bayesian phylogram of *Drosophila polo*-like genes. Numbers are posterior credibility values.

Polo is a protein kinase that, in *Drosophila* female meiosis promotes nuclear envelope breakdown [26]. Three *polo* genes are found in the two closely related species *D. pseudoobscura* and *D. persimilis* (Fig. 6). The *D. persimilis GL25129* and the *D. pseudoobscura GA11545* genes that are on Muller's element D (where the *D. melanogaster polo* gene is also located) are orthologous. The *D. persimilis GL25881* and the *D. pseudoobscura GA25172* genes that are on Muller's element B are also orthologous, and are here named *polo-dup1*. The *D. persimilis GL19429* and the *D. pseudoobscura GA25958* genes that are on Muller's element B are also orthologous and are here named *polo-dup2*.

There are three predicted introns in *polo-dup1*. Therefore, retrotransposition seems an unlikely explanation for the observed change in gene location (from Muller's element D to element B). This *polo* gene duplication is about 6.5 million years old (under the assumption of a molecular clock for synonymous mutations; data not shown), and is thus expected to be found in species of the *obscura* group only. It should be noted that the two *polo* genes are subjected to similar mutation rates but different amino acid constraints (Table 1). This observation could suggest that *polo-dup1* is a pseudogene. Nevertheless, *polo-dup1* is expressed in males (Fig. 4).

*polo-dup2* is about 12 million years old (under the assumption of a molecular clock for synonymous mutations; data not shown), and thus is also expected to be found in species of the *obscura* group only. There are no introns in *polo-dup2*. Therefore, in this case, retrotransposition could be an explanation for the origin of this duplication. It should be noted that such an hypothesis relies on the quality of the annotation of the *D. pseudoobscura* and *D. persimilis* genomes. *polo* and *polo-dup2* are subjected to similar mutation rates but different amino acid constraints (Table 1). Nevertheless, *polo-dup2* is expressed in males (Fig. 4), and thus, is unlikely to be a pseudogene.

There is one, three, and one fixed amino acid changes between the two *polo-dup1* gene sequences and the other *polo* sequences here analyzed, at the first, second and third Polo boxes, respectively. In general, it is difficult to infer how important these changes might be. It should be noted, however, that the amino acid change observed in Polo box 1 (a change of a V to a I) changes an amino acid that is conserved in *polo* sequences from fungi to humans (see Fig. 1 in [27]). The *polo-dup2* gene is a truncated version of *polo* where the last one third of the coding region of the gene is missing. Therefore the protein encoded by *polo-dup2* does not show any POLO boxes.

### Two independent *mtrm* gene duplications

The *D. melanogaster* Mtrm protein is a meiosis-specific 1∶1 stoichiometric inhibitor of the Polo kinase protein [28]. This gene is not annotated in most *Drosophila* genomes (Table S3) but can be always found within one intron of the *exo70* gene.

**Figure 7:**
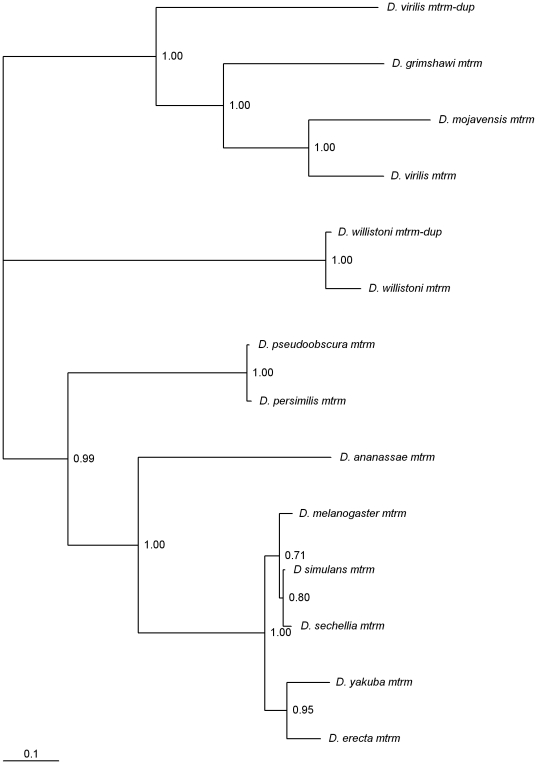
Bayesian phylogram of *Drosophila mtrm*-like genes. Numbers are posterior credibility values.

In *D. willistoni* there are two *mtrm*-like genes, (Fig. 7), one on Muller's element B (that codes for a 186 amino acids long protein) and another one on Muller's element D (that codes for a 196 amino acids long protein). Since the *D. melanogaster mtrm* gene is located on Muller's element D, it seems likely that the duplicated gene copy is that on Muller's element B, and thus this *D. willistoni* copy is here named *mtrm-dup*. Although the two copies are on different Muller's elements, at the nucleotide level, the two sequences are 94% identical. Since *mtrm* gene does not have introns the possible involvement of retrotransposition in the translocation of the gene cannot be assessed. This is a recent gene duplication event, estimated to be 1.6 million years old, under the assumption of a molecular clock for synonymous sites. The two genes seem to be under similar amino acid constraint (Table 1). In *D. melanogaster,* phosphorylation sites (including one Polo-box domain binding motif and one Plk-phosphorylation motif, that differs at one amino acid site from the canonical sequence D/E-X-S/T-Ø-X-D/E where Ø is an hydrophobic amino acid), have been reported [28]. Both *D. willistoni mtrm* genes show a Polo-box domain binding motif and a typical Plk-phosphorylation motif in the same protein region as in *D. melanogaster* (Table 2). Moreover, most of the other phosphorylation sites reported for the *D. melanogaster* Mtrm protein are also present in the *D. willistoni* Mtrm and Mtrm-dup proteins. The only phosphorylation site that is not present is also not conserved in Mtrm proteins from other *Drosophila* species. Nevertheless, we could not find any evidence that the *D. willistoni mtrm-dup* is expressed (Fig. 4). Therefore, the hypothesis that this gene is a recent pseudogene that did not have yet time to degenerate cannot be ruled out.

**Table 2:** Mtrm phosphorylation sites [28].

Species	T40 (STP)	S48	S52	S121	S123	S124	S132	S137
*D. melanogaster mtrm*	+	+	+	+	+	+	+	+
*D. simulans mtrm*	+	+	+		+	+	+	+
*D. sechellia mtrm*	+	+	+		+	+	+	+
*D. yakuba mtrm*	+	+	+		+	+	+	+
*D. erecta mtrm*	+	+	+		+	+	+	+
*D. ananassae mtrm*	+	+	+	+	+		+	+
*D. pseudoobscura mtrm*	+	+	+	+	+	+		+
*D. persimilis mtrm*	+	+	+	+	+	+		+
*D. willistoni mtrm*	+	+	+		+	+	+*	+
*D. willistoni mtrm-dup*	+	+	+		+	+	+*	+
*D. grimshawi mtrm*	+	+	+			+		+
*D. mojavensis mtrm*	+	+	+	+	+	+		+
*D. virilis mtrm*	+	+	+	+		+		+
*D. virilis mtrm-dup*	+	+	+			+		+
*D. lummei mtrm-dup*	+	+	+			+		+
*D. novamexicana mtrm-dup*	+	+	+			+		+
*D. americana texana mtrm-dup*	+	+	+			+		+
*D. americana americana mtrm-dup*	+	+	+			+		+
*D. littoralis mtrm-dup*	+	+	+			+		+
*D. kanekoi mtrm-dup*	+	+	+			+		+
*D. ezoana mtrm-dup*	+	+	+			+		+
*D. borealis Western mtrm-dup*	+	+	+			+		+
*D. flavomontana mtrm-dup*	+	+	+			+		+
*D. lacicola mtrm-dup*	+	+	+			+		+
*D. montana mtrm-dup*	+	+	+			+		+
*D. borealis Eastern mtrm-dup*	+	+	+			+		+

The referred amino acid positions are those of the *D. melanogaster* Mtrm sequence.

*in agreement with the D/E-X-S/T-Ø-X-D/E pattern where Ø is a hydrophobic amino acid.

In *D. virilis* there are also two *mtrm*-like genes, namely, one on Muller's element A and another one on Muller's element D, being the latter the orthologous of the *D. melanogaster mtrm* gene. *mtrm* and *mtrm-dup* are intronless genes. Therefore, it is not possible to infer the role of retrotransposition in the transposition of this gene from Muller's element D to A.

Bayesian phylogenetic analyses suggest that this *mtrm* gene duplication predates the separation of the *D. grimshawi*/(*D. mojavensis*/*D. virilis*) lineages (Fig. 7), and this conclusion is independent of the alignment algorithm used (data not shown). Moreover, *mtrm-dup* is not evolving faster than the *mtrm* gene (Table 1). The pair-wise synonymous divergence values suggest that, under the assumption of a molecular clock, *mtrm-dup* is about 35 million years old, and as such, would indeed predate the separation of the *D. grimshawi*/(*D. mojavensis*/*D. virilis*) lineages. Nevertheless, there is no evidence for a *mtrm* gene duplicate in either *D. grimshawi* or *D. mojavensis*. Therefore, taken at face value, these results imply two independent losses of the *mtrm-dup* gene.

The two neighbors of the *D. virilis mtrm-dup* gene (the *D. melanogaster CG7326* and *CG34401* orthologous genes) are gene neighbors in *D. grimshawi* and *D. mojavensis*. Each independent gene loss should be a unique event and thus leave a different genomic signature. Therefore, the comparison of the *CG7326* - *CG34401* region in *D. virilis, D. grimshawi* and *D. mojavensis* could, in principle, shed light on this issue. The intergenic regions can be confidently aligned, as revealed by the per site rates of change, namely 0.36 and 0.54 for the *D. virilis* – *D. mojavensis* and the *D. virilis* – *D. grimshawi* comparisons, respectively. The largest insertion, besides the *mtrm* coding region, in *D. virilis* relative to the other two species is only 27 bp long, and in total, there are 61 fixed gapped positions between the *D. virilis* sequence and the *D. grimshawi*/*D. mojavensis* sequences. Therefore, it seems that the only main difference in *D. virilis* relative to the other species is the insertion of the *mtrm* coding region. *mtrm-dup* is not, however, a pseudogene, since this gene is expressed in females (Fig. 4).

The *mtrm-dup* gene could also be amplified from 12 species of the *virilis* group from all major group phylads. Therefore, the *mtrm-dup* gene must be older than the age of the *virilis* group that is estimated to be 10 million years old [29]. Although 90% of the coding region of this gene was analyzed in the 12 species of the *virilis* group, no evidence for in-frame stop codons has been found. All *mtrm-dup* sequences show conservation of the T40 (a putative Cdk5 phosphorylation site), S48, S52 (putative Cdk or MAPK phosphorylation sites), S137, and S124 phosphorylation orthologous sites identified in *D. melanogaster* by Xiang *et al.* [28]. The S121 and S123 phosphorylation sites are not conserved in the *mtrm-dup* gene. Nevertheless, not all *mtrm* sequences show conservation of these sites either (Table 2).

The *mtrm-dup* gene does not show a Plk phosphorylation-like amino acid motif, due to a four amino acid insertion that is present in all *mtrm-dup* copies. It should be noted, however, that the *D. virilis*, *D. mojavensis* and *D. grimshawi mtrm* amino acid sequences do not have such a feature either, due to a three amino acid insertion. Therefore, in species of the *Drosophila* subgenus, the presence of a Plk phosphorylation-like amino acid motif is not an essential feature.

Although *mtrm-dup* is a functional gene, there are no data to support the assumption that this gene plays an essential role in meiosis in species of the *virilis* group of *Drosophila*. Indeed, it is conceivable that this gene represents a non-essential meiotic drive element that went to fixation in the common ancestor of species of the *virilis* group. Once fixed, it may be difficult to lose such an element since chromosomes carrying it are more represented in the next generation than chromosomes carrying alternative deleted copies of this element. Thus, such a gene could show most of the features expected for an essential gene. For *D. melanogaster*/*D. simulans* Anderson *et al.* [15] showed patterns of evolution at the *mtrm* gene that are compatible with both adaptive protein evolution and the sequential fixation of meiotic drive elements. Therefore, this hypothesis is here addressed in *D. americana*, a species of the *virilis* group of *Drosophila*.

Vieira *et al.* [17] reported an amino acid polymorphism for *D. americana*, at the gene *CG18543* (*mtrm-dup*) that is a marker for the common polymorphic *X*/*4* fusion. We have followed the transmission of the two types of chromosomes by looking at the male progeny of females heterozygous for the *mtrm-dup* amino acid variant under different genomic backgrounds (Table 3). There is no evidence that the reported amino acid polymorphism represents meiotic drivers of different strength (Chi-square test with one degree of freedom; P>0.05).

**Table 3:** Segregation of the common *D. americana mtrm-dup* amino acid polymorphism that is a marker for *X*/*4* fusion chromosomes.

Crosses	♀x♂		♂x♀	
	*X*/*4* fusion	Non-fusion	*X*/*4* fusion	Non-fusion
NN97.4 × W29	8	2	5	5
NN97.4 × LP97.7	5	5	6	4
NN97.4 × ML97.4.2	6	4	6	2
NN97.4 × ML97.5	4	6	6	4
NN97.8 × W29	1	9	7	3
NN97.8 × LP97.7	10	0	8	2
NN97.8 × ML97.4.2	4	6	5	5
NN97.8 × ML97.5	6	4	8	2
W11 × W29	7	3	6	4
W11 × LP97.7	6	4	7	3
W11 × ML97.4.2	5	5	4	6
W11 × ML97.5	7	3	6	4
W23 × W29	3	6	2	8
W23 × LP97.7	3	7	6	4
W23 × ML97.4.2	3	7	6	4
W23 × ML97.5	5	5	2	8
Total	83	76	90	68

### Concerted evolution at the *Drosophila* subgenus *meiS332*-like genes

**Figure 8:**
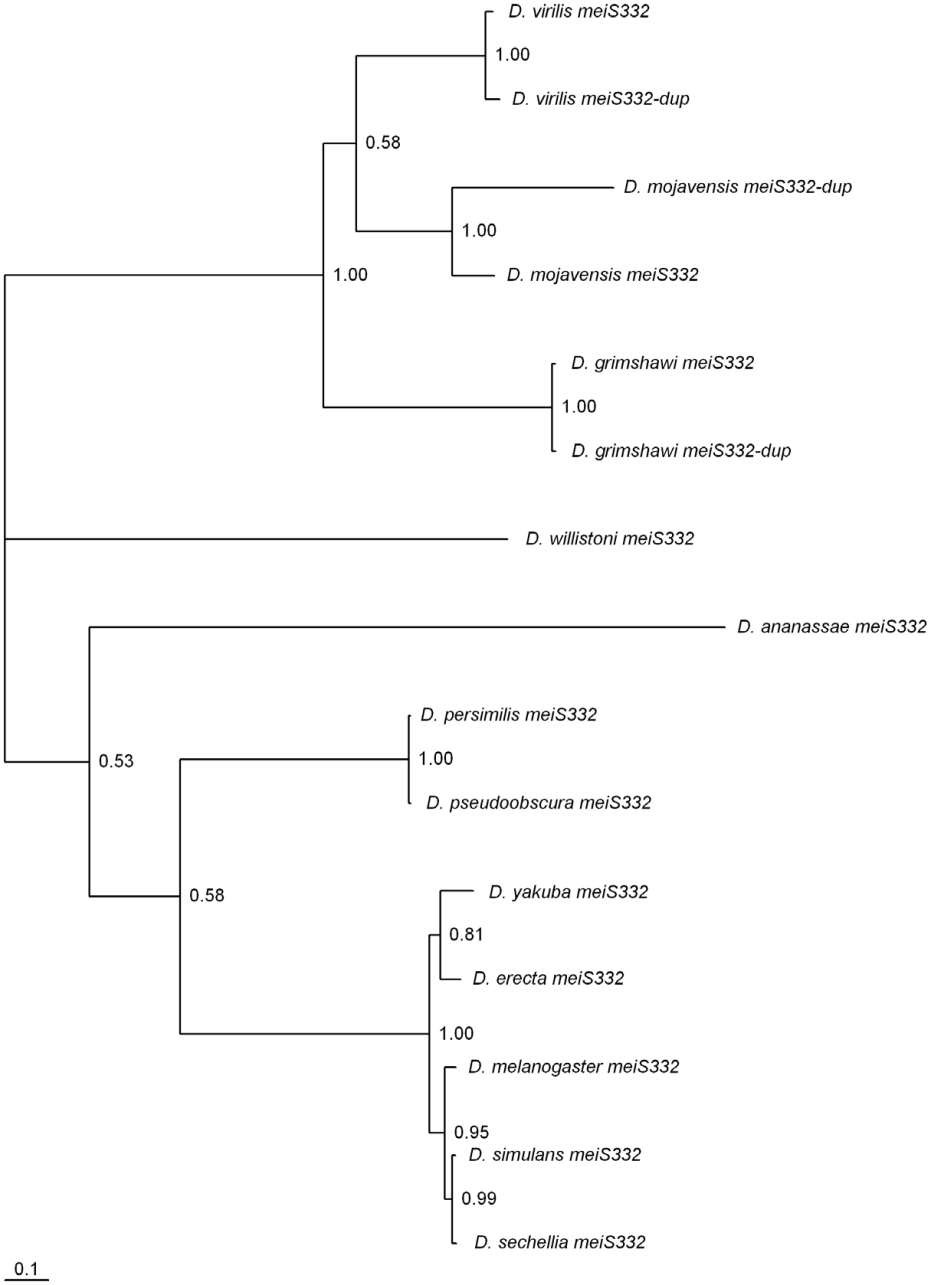
Bayesian phylogram of *Drosophila meiS332*-like genes. Numbers are posterior credibility values.

*meiS332* gene duplications have been found as well. The phylogeny presented in Fig. 8 suggests that this gene has been independently duplicated three times. Nevertheless, the two copies of the gene are located on Muller's element C always with opposite transcription orientations, and at about the same distance. The finding of a similar gene arrangement in *D. virilis*, *D. mojavensis* and *D. grimshawi* thus suggests a unique duplication event, rather than three independent recent duplications. The little divergence observed between the two copies in each species suggests that this is a case of concerted evolution. Concerted evolution has been reported at *Drosophila* genes other than rRNA gene loci (see for instance [4], [30], [31], [32], [33], [34]. The *meiS332* gene duplication is an example of long-term (more than 30 million years) concerted evolution in the *Drosophila* subgenus. Similar long-term concerted evolution (also lasting for more than 30 million years) has been reported at the *polyhomeotic* (*ph*) gene duplication in the *Sophophora* subgenus [32].

In *D. melanogaster,* there are two Polo binding sites in MEI-S332, namely SSP from residue 233 to 235, and STP from residue 330 to 332 [35]. As shown in Table 4, the SSP motif is conserved in species of the *melanogaster* subgroup, in the two *D. grimshawi* sequences and in one of the two *D. virilis* sequences. The STP motif is conserved in all sequences with the exception of the *D. mojavensis* duplicated copy. It should be noted that, in *D. melanogaster,* phosphorylation was unaffected by the S234A mutation but was abolished with the T331A mutation [35]. This finding fits our observation of a better conservation of the STP motif than that of the SSP motif. Most MEI-S332 sequences from species of the *Drosophila* subgenus show three S(S/T)P motifs. These findings suggest that, with the exception of the *D. mojavensis* duplicate, all other duplicated genes are functional. Nevertheless, expression was detected in *D. virilis* and *D. mojavensis meiS332-dup* gene. We could not obtain non-mutant *D. grimshawi* strains, and thus expression was not tested in this species.

**Table 4:** MeiS332 Polo binding sites (SSP and STP).

Species	Motif and amino acid site reference(position in the *D. melanogaster* sequence)				
	SSP150	SSP185	SSP233	SSP302	STP330
*D. melanogaster meiS332*			+		+
*D. simulans meiS332*			+		+
*D. sechellia meiS332*			+		+
*D. yakuba meiS332*			+		+
*D. erecta meiS332*			+		+
*D. ananassae meiS332*					+
*D. pseudoobscura meiS332*					+
*D. persimilis meiS332*					+
*D. willistoni meiS332*					+
*D. grimshawi meiS332*		+	+		+
*D. grimshawi meiS332-dup*		+	+		+
*D. mojavensis meiS332*		+		+	+
*D. mojavensis meiS332-dup*					
*D. virilis meiS332*		+	+		+
*D. virilis meiS332-dup*	+				+

## Discussion

Nine independent gene duplications involving the genes *cav*, *mre11*, *meiS332*, *polo* and *mtrm* were found. The 12 *Drosophila* species here analyzed imply about 230 million years of independent evolution. Therefore, *Drosophila* meiosis-related genes are duplicated and retained at a rate of 0.0012 per gene per million years. This value is similar to that estimated for the whole *Drosophila* genome using species of the *melanogaster* subgroup [1]. The rate at which gene duplicates are created and go to fixation, i.e, are retained, depends on population genetics variables such as birth rate, mutation rate, and effective population size (see for instance, [36]). While it is unlikely that those population genetics variables have remained constant over 230 million years of independent evolution, there is no reason to believe that using all 12 *Drosophila* genomes and all genes would produce an estimate that is substantially different from that provided by Osada and Innan [1]. For instance, when the dataset of 33 meiosis related genes is used, the rate of gene duplication and fixation is estimated to be 0.0013 and 0.0011 for species of the *Drosophila* and *Sophophora* subgenera, respectively (the estimate becomes 0.0009 for the *Sophophora* subgenus if the only likely non-functional *D. willistoni mtrm-dup* gene is not included in the calculations; see Table 5). It should be noted that, a detailed analysis of the 33 meiosis genes, revealed that a substantial fraction is non-annotated or likely miss-annotated. Although we do not provide a human-curated annotation for the studied genes in the 12 *Drosophila* genomes, we did analyze in detail the gene annotation for those cases where the non-annotation or miss-annotation could lead to erroneous conclusions (see Results).

**Table 5:** Summary of the inferences made for the meiosis genes found to be duplicated.

Duplicated gene copy	Location (Muller's element)	Estimated age in million years	Comments
*D. persimilis* and *D. pseudoobscura cav-dup*	A	∼40	Likely functional
*D. willistoni cav-dup*	B	10	Likely functional
*D. virilis cav-dup*	E	14	Likely functional
*D. mojavensis mre11-dup*	B	15	Likely functional
*D. persimilis* and *D. pseudoobscura polo-dup1*	B	6.5	Likely functional
*D. persimilis* and *D. pseudoobscura polo-dup2*	B	12	Likely functional
*D. willistoni mtrm-dup*	B	1.6	Likely non-functional
*D. virilis mtrm-dup*	A	∼35	Likely functional
*D. grimshawi*, *D. mojavensis* and *D. virilis meiS332-dup*	C	> 30	Likely functional

The finding that functional meiosis-related gene duplications go to fixation at the same rate as the average for all genes is surprising, especially in the light of the complex roles that the genes under study perform (see Table S1). Indeed, meiosis-related genes are known to participate in multiple pathways, be involved in protein complexes, and, when disrupted, affect multiple aspects of meiosis (see Table S1).

It remains to be shown whether the gene duplicates play an essential role in meiosis-related features in the species where they are found. Therefore, it could be argued that they are non-essential meiotic drive gene duplicates that went to fixation. Nevertheless, the segregation experiments performed with the *D. americana mtrm-dup* gene did not reveal evidence for meiotic drive elements. The possibility of subfunctionalization [37] cannot be, however, ruled out. In *Arabidopsis*, gene duplicates involved in DNA repair, replication and recombination, as well as in cell-cycle are little retained [11].

The possibility that the duplicated meiosis related genes represent cases of neofunctionalization should thus be addressed by performing additional detailed cellular and biochemical experiments that are beyond the scope of this work. Indeed, about 50% of the gene duplicates are evolving faster than the original gene, a pattern that is compatible with a short period of relaxed selection or/and acquisition of a new function. Moreover, three out of the five genes that have been found to be duplicated are known to physically interact (*meiS332*, *polo* and *mtrm*). There are no reasons to believe that these genes are more prone to accumulate meiotic drive elements or more prone to subfunctionalization. Indeed, given the known function of these genes, they were, *a priori*, unlikely to be found duplicated. The *D. melanogaster* Mtrm protein is a meiosis-specific 1∶1 stoichiometric inhibitor of the Polo kinase protein. In this species activation of Cdc25 by an excess of Polo protein at stage 13 triggers nuclear envelope breakdown and entry into prometaphase [28]. Therefore, any changes in protein levels in either Polo or Mtrm could result in precocious entry into prometaphase or meiotic arrest. On the other hand, Polo antagonizes MeiS332 and removes this protein from centromeres, a step required for proper chromosome segregation at the metaphase II/anaphase II transition [35]. If meiosis is not completed, no gametes will be produced. On the other hand, significant defects in achiasmate segregation (the segregation of chromosomes that did not experience recombination) are observed when there is a precocious entry into prometaphase [28]. Therefore, in what follows we speculate on the conceivable adaptive value of each gene duplicate(s).

cav is a DNA-binding protein that is a component of the multiprotein *Drosophila* origin recognition complex [24]. In *Drosophila*, the *cav* gene has been duplicated three times independently. All three independent duplications are old and all *cav* duplicates are expressed. The functional significance of having two *cav* gene copies in *D. virilis* with similar expression patterns is unclear, but it could be related to the high *D. virilis* heterochromatin content. Although *D. melanogaster* and *D. virilis* have similar euchromatin sizes, the C- value for these species is about 0.17 and 0.37, respectively (http://www.genomesize.com). It has been proposed that heterochromatin protein 1 (HP1), in association with the origin recognition complex, recruits underphosphorylated isoforms of HP1 to sites of heterochromatin nucleation [38]. High cav-related protein levels could be advantageous in species with high heterochromatin content such as *D. virilis*.

The functional significance of having in *D. persimilis*/*D. pseudoobscura* a *cav* gene duplicate is also unclear. Even more puzzling is the functional significance of having one *cav* gene duplicate in *D. willistoni* with an apparent male-specific expression, since the original *cav* gene is expressed in both females and males. It should be noted that the C-value of these species is similar to the one reported for *D. melanogaster* (http://www.genomesize.com). Detailed expression studies are needed in order to address this issue.

The Mre11 protein is involved in telomere maintenance by preventing telomere fusion [25], [39]. In *D. mojavensis* there are two *mre11*-like genes. *mre11* is expressed both in males and females being, however, more highly expressed in males. The *mre11-dup* gene seems to be expressed in males only. Therefore, in principle, the effect of the gene duplication is to exacerbate even more the difference in Mre11 expression levels in males and females. It can be speculated that *D. mojavensis* telomeres are for some reason stickier than those of other species. This is a possibility because the telomeric and half-telomeric retrotransposons of *D. mojavensis* display a number of unique features when compared to other *Drosophila* species [40]. In *D. melanogaster*, as in any eukaryote, recombination-based mechanisms also help maintain chromosome termini [41]. Nevertheless, in *Drosophila* males, there is no recombination, and thus the higher *mre11* expression levels in males than in females might have been anticipated.

Two functional *polo* gene duplicates are observed in *D. persimilis*/*D. pseudoobscura*. *polo-dup1* and *polo-dup2* are apparently exclusively expressed in males. It can thus be predicted that in the *obscura* group of *Drosophila* nuclear envelope breakdown and entry into prometaphase occurs earlier in males from these species when compared with what happens in *D. melanogaster*.

The *D. melanogaster* Mtrm protein is a meiosis-specific 1∶1 stoichiometric inhibitor of the Polo kinase protein. Two independent duplications of this gene were found, one in *D. willistoni* and the other in *D. virilis*. The *D. willistoni mtrm-dup* gene seems to be a recent pseudogene, whereas strong evidence is here presented supporting the fact that the *D. virilis mtrm-dup* is an old functional gene duplication. It is unlikely that *mtrm-dup* is a meiotic drive element that was duplicated just by chance. It can thus be predicted that in *D. virilis* nuclear envelope breakdown and entry into prometaphase occurs later than in *D. melanogaster*. It should be noted that the *D. virilis mtrm-dup* is expressed in females only.

There are functional gene duplicates of *meiS332* in *D. mojavensis* and *D. virilis*. If there is more MeiS332 protein to be removed from centromeres by Polo, then meiosis would be delayed, since removal of MeiS332 from centromeres is a step required for proper chromosome segregation at the metaphase II/anaphase II transition. Interestingly, in *D. virilis* females the *mtrm* gene is also duplicated. As noted above, an increase in Mtrm protein levels is also predicted to result in a delay in meiosis. A delayed meiosis could result in more time available to deal with large genomes such as that of *D. virilis*. It is, however, unclear whether the high heterochromatin content found in *D. virilis* is the consequence of an historically advantageous long meiosis duration that allowed the accumulation of high amounts of heterochromatin without deleterious consequences, or whether the long meiosis duration is an adaptive response aiming at handling the large amount of heterochromatin found in this species, that may have accumulated due to other reasons.

In conclusion, in this work we find that, contrary to theoretical expectations, meiosis-related genes are duplicated and retained at the same rate as the average for all genes. The duplicated genes were, *a priori*, unlikely to be found duplicated, and may represent examples of neofunctionalization. Detailed cellular and biochemical experiments must be performed in order to address this issue. Nevertheless, given the nature of the genes that were found duplicated, it is here speculated that the duplicated genes may affect meiosis duration. *D. melanogaster* is the only *Drosophila* species where meiosis duration has been recorded (it takes about 1-2 days; [13]). The results here presented suggest that in the *obscura* group of species, male meiosis duration may be shorter than in *D. melanogaster*, while in *D. virilis,* where three meiosis genes are duplicated, meiosis duration may be much longer than in *D. melanogaster*. Interestingly, *D. virilis* is among the *Drosophila* species the one with highest nuclear DNA content, and Bennett [13] has shown a linear correlation in insects between nuclear DNA content and the duration of meiosis. If the correlation derived by Bennett holds true, then, at the same temperature, meiosis should take about twice as long in *D. virilis* than in *D. melanogaster*. Environmental factors should be taken into consideration as well, when making such predictions. Indeed, Bennett [13] shows that in insects, a decrease of 10°C in environmental temperature means a doubling in meiosis duration. Therefore, under their natural environments, *Drosophila* temperate species (such as species of the *virilis* group) should show, anyway, longer meiosis duration times than tropical species (such as *D. melanogaster* African populations).

## Supporting Information

Table S1 **Overview of the meiosis-related genes studied.**
(PDF)

Table S2 **List of primers used.**
(PDF)

Table S3 **Accession numbers for the 33 meiosis genes studied from 12** ***Drosophila*** **species.**
(PDF)

Table S4 **Coding sequence size and intron number (in brackets) of 33 meiosis genes from 12** ***Drosophila*** **species.**
(PDF)
