# Supplementary material for: Drosophila Genes That Affect Meiosis Duration Are among the Meiosis Related Genes That Are More Often Found Duplicated
Source: PLoS One. 2011 Mar 10;6(3):e17512. doi: 10.1371/journal.pone.0017512 (PMC3053365; doi:10.1371/journal.pone.0017512)
Supplement: Table S1 — Overview of the meiosis-related genes studied. (PDF) [file pone.0017512.s001.pdf]

Table S1. Overview of the meiosis-related genes studied.

Gene	Function	References
polo	involved in the timing control of meiotic prophase I entry, the restriction of meiosis to the oocyte and the initiation/maintenance of SC required to activate Twine, initiating the chain of events that leads to GVBD and prometaphase I; at meiosis II, Polo is essential for the phosphorylation and removal of MEI-S332	[1,2,3]
matrimony	stoichiometric inhibitor of Polo until stage 13 of oocyte development	[1]
c(2)M [mei-910]	part of the SC; required for DSBs to be repaired as crossovers	[4]
c(3)G	part of the SC; meiotic recombination	[5,6,7]
CG7676 [corona]	normal C(3)G localization; assembly of the central region of SC	[8]
mei-P22	DSBs formation	[9]
mei-W68	DSBs formation	[10,11]
mei-41 [ATR]	part of the DDR pathway; DNA-dependent protein kinase required for checkpoint response to DSBs	[12,13]
mus304 [ATRIP]	part of the DDR pathway; required for chromosome break repair and for genomic stability; acts in the mei-41 pathway	[14]
ald [Mps1]	part of the SAC pathway; meiotic and mitotic spindle assembly checkpoint	[15,16]
okr [rad54]	stimulate and regulate meiotic DSBs repair process	[17,18]
spn-B	strand-exchange protein in the DSBs repair	[18]
spn-A [Rad51]	enzyme in DNA repair, mediating the strand invasion and strand exchange steps of HR	[19]
spn-D	might encode proteins that act in recombinational repair of DSBs	[18,20,21]

mei-218	determine which DSBs sites will be repaired as crossovers; maybe part of a pathway linking germline differentiation and the regulation of meiotic recombination events	[22,23,24]
mei-9	required for resolution of Holliday junctions after DSBs repair	[25]
mei-P26	oocyte differentiation	[26]
ncd	spindle pole formation	[27,28]
subito [Dub, mei-1794]	meiotic spindle pole formation	[29]
asp	MT-binding protein that binds to the minus ends of spindle MTs and cross-links them into focused structures	[30]
Klp3A [mei-352]	in mitosis Feo/Klp3A complex is necessary for Polo recruitment to the spindle midzone; involved in the organization of interpolar microtubules and in anaphase B spindle elongation during mitosis; in female meiosis is specifically required for mediating the distribution of exchanges; in male meiosis is necessary for contractile ring assembly during cytokinesis; involved in the separation of the female pronucleus from the polar bodies or in the migration of the female pronucleus toward the male pronucleus	[31,32,33,34,35,36]
mei-S332 [Shugoshin]	essential in meiosis for maintaining cohesion at centromeres until sister chromatids separate at the metaphase II/anaphase II transition	[37,38]
ord	sister chromatid cohesion; required for the maintenance of chromosome core integrity during meiotic prophase; promote homologue bias during meiotic recombination	[39,40]
SMC1	part of the cohesin complex	[41,42]
Axs	normal separation of paired achiasmate homologs	[43]
teflon	required in males for proper segregation of autosomes	[44]
tefu [ATM]	regulate levels of HP1 and HOAP at telomeres; prevent chromosome end fusions; promote telomere-position effect	[45]

mre11	part of the MRN complex; DNA repair protein; telomere localization of HP1 and HOAP	[46,47,48]
rad50	part of the MRN complex; DNA repair protein; telomere localization of HP1 and HOAP	[46,47,48]
cav [HOAP]	telomere-protection; gene silencing at pericentric heterochromatin; telomere capping	[49]
Su(var)205 [HP1]	telomere-protection; acts as negative regulators of telomere length; telomere capping;	[49,50,51]
Ku70 [Irbp]	part of the Ku70/80 heterodimer; acts as negative regulators of telomere length; control telomere elongation	[52]
Ku80	part of the Ku70/80 heterodimer; acts as negative regulators of telomere length; control telomere elongation	[52]

Common gene synonyms are indicated enclosed by square brackets. Abbreviations used: SC - Synaptonemal complex; GVBD - Germinal vesicle breakdown;

DSBs - Double-strand breaks; DDR - DNA damage response; SAC - spindle assembly checkpoint; HR - Homologous recombination; MT - microtubule

References

1. Xiang Y, Takeo S, Florens L, Hughes SE, Huo LJ, *et al.* (2007) The inhibition of polo kinase by matrimony maintains G2 arrest in the meiotic cell cycle. *PLoS Biol* 5: e323.

2. Mirouse V, Formstecher E, Couderc JL (2006) Interaction between Polo and BicD proteins links oocyte determination and meiosis control in *Drosophila*. *Development* 133: 4005-4013.

3. Clarke AS, Tang TT, Ooi DL, Orr-Weaver TL (2005) POLO kinase regulates the *Drosophila* centromere cohesion protein MEI-S332. *Dev Cell* 8: 53-64.

4. Manheim EA, Jang JK, Dominic D, McKim KS (2002) Cytoplasmic localization and evolutionary conservation of MEI-218, a protein required for meiotic crossing-over in *Drosophila*. *Mol Biol Cell* 13: 84-95.

5. Carlson PS (1972) The effects of inversions and the *C(3)G* mutation on intragenic recombination in *Drosophila*. *Genet Res* 19: 129-132.

6. Hall JC (1972) Chromosome segregation influenced by two alleles of the meiotic mutant *c(3)G* in *Drosophila melanogaster*. *Genetics* 71: 367-400.

7. Page SL, Hawley RS (2001) *c(3)G* encodes a *Drosophila* synaptonemal complex protein. *Genes Dev* 15: 3130-3143.

8. Page SL, Khetani RS, Lake CM, Nielsen RJ, Jeffress JK, *et al.* (2008) Corona is required for higher-order assembly of transverse filaments into full-length synaptonemal complex in *Drosophila* oocytes. *PLoS Genet* 4: e1000194.

9. Liu H, Jang JK, Kato N, McKim KS (2002) *mei-P22* encodes a chromosome-associated protein required for the initiation of meiotic recombination in *Drosophila melanogaster*. *Genetics* 162: 245-258.

10. McKim KS, Hayashi-Hagihara A (1998) *mei-W68* in *Drosophila melanogaster* encodes a Spo11 homolog: evidence that the mechanism for initiating meiotic recombination is conserved. *Genes Dev* 12: 2932-2942.

11. Keeney S, Giroux CN, Kleckner N (1997) Meiosis-specific DNA double-strand breaks are catalyzed by Spo11, a member of a widely conserved protein family. *Cell* 88: 375-384.

12. Hari KL, Santerre A, Sekelsky JJ, McKim KS, Boyd JB, *et al.* (1995) The *mei-41* gene of *D. melanogaster* is a structural and functional homolog of the human ataxia telangiectasia *gene*. *Cell* 82: 815-821.

13. Roeder GS (1997) Meiotic chromosomes: it takes two to tango. *Genes Dev* 11: 2600-2621.

14. Brodsky MH, Sekelsky JJ, Tsang G, Hawley RS, Rubin GM (2000) *mus304* encodes a novel DNA damage checkpoint protein required during *Drosophila* development. *Genes Dev* 14: 666-678.

15. Gilliland WD, Hughes SE, Cotitta JL, Takeo S, Xiang Y, *et al.* (2007) The multiple roles of mps1 in *Drosophila* female meiosis. *PLoS Genet* 3: e113.

16. Page SL, Nielsen RJ, Teeter K, Lake CM, Ong S, *et al.* (2007) A germline clone screen for meiotic mutants in *Drosophila melanogaster*. *Fly* (Austin) 1: 172-181.

17. Kooistra R, Vreeken K, Zonneveld JB, de Jong A, Eeken JC, *et al.* (1997) The *Drosophila melanogaster* RAD54 homolog, DmRAD54, is involved in the repair of radiation damage and recombination. *Mol Cell Biol* 17: 6097-6104.

18. Ghabrial A, Ray RP, Schupbach T (1998) *okra* and *spindle-B* encode components of the RAD52 DNA repair pathway and affect meiosis and patterning in *Drosophila* oogenesis. *Genes Dev* 12: 2711-2723.

19. Staeva-Vieira E, Yoo S, Lehmann R (2003) An essential role of DmRad51/SpnA in DNA repair and meiotic checkpoint control. *Embo J* 22: 5863-5874.

20. Ghabrial A, Schupbach T (1999) Activation of a meiotic checkpoint regulates translation of Gurken during *Drosophila* oogenesis. *Nat Cell Biol* 1: 354-357.

21. Boyd JB, Harris PV (1981) Mutants partially defective in excision repair at five autosomal loci in *Drosophila melanogaster*. *Chromosoma* 82: 249-257.

22. Carpenter AT (1982) Mismatch repair, gene conversion, and crossing-over in two recombination-defective mutants of *Drosophila melanogaster*. *Proc Natl Acad Sci USA* 79: 5961-5965.

23. Carpenter AT (1984) Recombination nodules and the mechanism of crossing-over in *Drosophila*. *Symp Soc Exp Biol* 38: 233-243.

24. McKim KS, Jang JK, Manheim EA (2002) Meiotic recombination and chromosome segregation in *Drosophila* females. *Annu Rev Genet* 36: 205-232.

25. Sekelsky JJ, McKim KS, Chin GM, Hawley RS (1995) The *Drosophila* meiotic recombination gene *mei-9* encodes a homologue of the yeast excision repair protein Rad1. *Genetics* 141: 619-627.

26. Page SL, McKim KS, Deneen B, Van Hook TL, Hawley RS (2000) Genetic studies of *mei-P26* reveal a link between the processes that control germ cell proliferation in both sexes and those that control meiotic exchange in *Drosophila*. *Genetics* 155: 1757-1772.

27. Hatsumi M, Endow SA (1992) The *Drosophila* ncd microtubule motor protein is spindle-associated in meiotic and mitotic cells. *J Cell Sci* 103 (Pt 4): 1013-1020.

28. Matthies HJ, McDonald HB, Goldstein LS, Theurkauf WE (1996) Anastral meiotic spindle morphogenesis: role of the non-claret disjunctional kinesin-like protein. *J Cell Biol* 134: 455-464.

29. Giunta KL, Jang JK, Manheim EA, Subramanian G, McKim KS (2002) *subito* encodes a kinesin-like protein required for meiotic spindle pole formation in *Drosophila melanogaster*. *Genetics* 160: 1489-1501.

30. Morales-Mulia S, Scholey JM (2005) Spindle pole organization in *Drosophila* S2 cells by dynein, abnormal spindle protein (Asp), and KLP10A. *Mol Biol Cell* 16: 3176-3186.

31. D'Avino PP, Archambault V, Przewloka MR, Zhang W, Lilley KS, *et al.* (2007) Recruitment of Polo kinase to the spindle midzone during cytokinesis requires the Feo/Klp3A complex. *PLoS One* 2: e572.

32. Brust-Mascher I, Civelekoglu-Scholey G, Kwon M, Mogilner A, Scholey JM (2004) Model for anaphase B: role of three mitotic motors in a switch from poleward flux to spindle elongation. *Proc Natl Acad Sci USA* 101: 15938-15943.

33. Kwon M, Morales-Mulia S, Brust-Mascher I, Rogers GC, Sharp DJ, *et al.* (2004) The chromokinesin, KLP3A, dives mitotic spindle pole separation during prometaphase and anaphase and facilitates chromatid motility. *Mol Biol Cell* 15: 219-233.

34. Williams BC, Riedy MF, Williams EV, Gatti M, Goldberg ML (1995) The *Drosophila* kinesin-like protein KLP3A is a midbody component required for central spindle assembly and initiation of cytokinesis. *J Cell Biol* 129: 709-723.

35. Williams BC, Dernburg AF, Puro J, Nokkala S, Goldberg ML (1997) The *Drosophila* kinesin-like protein KLP3A is required for proper behavior of male and female pronuclei at fertilization. *Development* 124: 2365-2376.

36. Giansanti MG, Bonaccorsi S, Williams B, Williams EV, Santolamazza C, *et al.* (1998) Cooperative interactions between the central spindle and the contractile ring during *Drosophila* cytokinesis. *Genes Dev* 12: 396-410.

37. Tang TT, Bickel SE, Young LM, Orr-Weaver TL (1998) Maintenance of sister-chromatid cohesion at the centromere by the *Drosophila* MEI-S332 protein. *Genes Dev* 12: 3843-3856.

38. Moore DP, Page AW, Tang TT, Kerrebrock AW, Orr-Weaver TL (1998) The cohesion protein MEI-S332 localizes to condensed meiotic and mitotic centromeres until sister chromatids separate. *J Cell Biol* 140: 1003-1012.

39. Bickel SE, Wyman DW, Miyazaki WY, Moore DP, Orr-Weaver TL (1996) Identification of ORD, a *Drosophila* protein essential for sister chromatid cohesion. *Embo J* 15: 1451-1459.

40. Webber HA, Howard L, Bickel SE (2004) The cohesion protein ORD is required for homologue bias during meiotic recombination. *J Cell Biol* 164: 819-829.

41. Hirano T (2002) The ABCs of SMC proteins: two-armed ATPases for chromosome condensation, cohesion, and repair. *Genes Dev* 16: 399-414.

42. Haering CH, Nasmyth K (2003) Building and breaking bridges between sister chromatids. *Bioessays* 25: 1178-1191.

43. Whyte WL, Irick H, Arbel T, Yasuda G, French RL, *et al.* (1993) The genetic analysis of achiasmate segregation in *Drosophila melanogaster*. III. The wild-type product of the *Axs* gene is required for the meiotic segregation of achiasmate homologs. *Genetics* 134: 825-835.

44. Tomkiel JE, Wakimoto BT, Briscoe A, Jr. (2001) The *teflon* gene is required for maintenance of autosomal homolog pairing at meiosis I in male *Drosophila melanogaster*. *Genetics* 157: 273-281.

45. Oikemus SR, McGinnis N, Queiroz-Machado J, Tukachinsky H, Takada S, *et al.* (2004) *Drosophila* atm/telomere fusion is required for telomeric localization of HP1 and telomere position effect. *Genes Dev* 18: 1850-1861.

46. Bi X, Wei SC, Rong YS (2004) Telomere protection without a telomerase; the role of ATM and Mre11 in *Drosophila* telomere maintenance. *Curr Biol* 14: 1348-1353.

47. Ciapponi L, Cenci G, Ducau J, Flores C, Johnson-Schlitz D, *et al.* (2004) The *Drosophila* Mre11/Rad50 complex is required to prevent both telomeric fusion and chromosome breakage. *Curr Biol* 14: 1360-1366.

48. Shiloh Y (2003) ATM and related protein kinases: safeguarding genome integrity. *Nat Rev Cancer* 3: 155-168.

49. Cenci G, Siriaco G, Raffa GD, Kellum R, Gatti M (2003) The *Drosophila* HOAP protein is required for telomere capping. *Nat Cell Biol* 5: 82-84.

50. Savitsky M, Kravchuk O, Melnikova L, Georgiev P (2002) Heterochromatin protein 1 is involved in control of telomere elongation in *Drosophila melanogaster*. *Mol Cell Biol* 22: 3204-3218.

51. Fanti L, Giovinazzo G, Berloco M, Pimpinelli S (1998) The heterochromatin protein 1 prevents telomere fusions in *Drosophila*. *Mol Cell* 2: 527-538.

52. Melnikova L, Biessmann H, Georgiev P (2005) The Ku protein complex is involved in length regulation of *Drosophila* telomeres. *Genetics* 170: 221-235.
