# Supplementary material for: Drosophila Genes That Affect Meiosis Duration Are among the Meiosis Related Genes That Are More Often Found Duplicated
Source: PLoS One. 2011 Mar 10;6(3):e17512. doi: 10.1371/journal.pone.0017512 (PMC3053365; doi:10.1371/journal.pone.0017512)
Supplement: Table S2 — List of primers used. (PDF) [file pone.0017512.s002.pdf]

Table S2. List of primers used.

Gene	Species	Primer_name	Primer_F (5' - 3')	Primer_name	Primer_R (5' - 3')	AT (ºC)	Size (bp)
RpL32	All except *D. willistoni* and *D. persimilis*	RpL32_virmojF	AATCTCCTTGCGTTTCTT	RpL32_virmojR	CACTTCATCCGTCACCAG	52	346
Rpl32	*D. willistoni* and *D. persimilis*	Rpl32_wil_perF	CTTCATCCGCCATCAGTC	Rpl32_wil_perF	GAACGCAGACGACCATTG	54	408
meiS332	*D. mojavensis*	Dmoj_meiS332_1F	TTATGTAGTCACGAAAGC	Dmoj_meiS332_1R	AGGAAGCCAAAGTATCAC	49	459
		Dmoj_meiS332_2F	TTGCTGGAGGAACGAGAA	Dmoj_meiS332_2R	ACGGACGAAGATGAGGTG	54	585
	D. virilis	Dvir_meiS332_1F	AGGCTGAGATATTACTGA	Dvir_meiS332_1R	TAGGTGACAAGGACAAAG	50	612
		Dvir_meiS332_2F	AGAAGCAACGAATAACTA	Dvir_meiS332_2R	TGAAGCAGCAACTGATGT	50	552
cav	*D. virilis*	Dvir_cav1F	GCCGATGAACTGGATGGT	Dvir_cav1R	TGCTGATGACTTTTGCTC	54	527
		Dvir_cav2F	CAAAATGAGGAGGAAGAA	Dvir_cav2R	GACACTGAGCCGAGCAAC	54	515
	D. willistoni	Dwil_cav1F	AAGAACAACCAGGATTTA	Dwil_cav1R	CGAAGGCACAGATACATT	51	609
		Dwil_cav2F	ATGTCGCTTTTGGATTCA	Dwil_cav2R	GGTTTTAGGTGGATTCTC	50	529
	D. persimilis	Dper_cav1F	AAATGGTGATGAGCAATG	Dper_cav1R	GAGAACGAGGTGGTGACA	52	483
		Dper_cav2F	GAGAAAGGCGAAAAATAC	Dper_cav2R	TCCCACACAGAGTTGAAG	51	220
mre11	*D. mojavensis*	Dmoj_mre11_1F	GTTGGCACTGTATGGATT	Dmoj_mre11_1R	AGCGGATTTTTGTCTTTG	51	869
		Dmoj_mre11_2F	ATCGTGGTGGTAGCAAGA	Dmoj_mre11_2R	GGAGTGAAAAAGCGTGAG	53	729
polo	*D. persimilis*	Dper_polo1F	CGGCAAAATCGTATCAAA	Dper_polo1R	GTCTTCAATGGTGTCGTT	53	831
		Dper_polo2F	AACGGTATTGAGCAGTCG	Dper_polo2R	TTCTTTTCCAGGGTCTTG	55	455
		Dper_polo3F	CCATTCCACCACAAAACC	Dper_polo3R	CCCAGTATTCCCATCTCC	57	429

mtrm	*D. virilis*	mtrm_aut_F	TTGACCGTGGACAGCATC	mtrm_aut_R	GTTTCTTGACAGGATTTG	51	190
		mtrm_amerF	TGGTCAGCATCTATTCAGC	mtrm_amerR	GCAGTCCTCATCATTACG	50	511
	D. willistoni	Dwil_mtrm1F	CACACCAACCAAAAATGA	Dwil_mtrm1R	AGTGCCTCCAGTATGATA	51	510<br>420
