# Supplementary material for: Drosophila Genes That Affect Meiosis Duration Are among the Meiosis Related Genes That Are More Often Found Duplicated
Source: PLoS One. 2011 Mar 10;6(3):e17512. doi: 10.1371/journal.pone.0017512 (PMC3053365; doi:10.1371/journal.pone.0017512)
Supplement: Table S3 — Accession numbers for the 33 meiosis genes studied from 12 Drosophila species. (PDF) [file pone.0017512.s003.pdf]

Table S3. Accession numbers for the 33 meiosis genes studied from 12 *Drosophila* species. When the gene is not annotated genomic coordinates are given for the gene fragment that can be found by Blast.

	D. melanogaster	*D. simulans*	*D. sechellia*	*D. yakuba*	*D. erecta*	*D.ananassae*	*D. pseudoobscura*	*D. persimilis*	*D. willistoni*	*D. mojavensis*	*D. virilis*	*D. grimshawi*
ald	CG7643	GD19180	GM15256	GE25491	GG22427	GF17824	GA20501	GL21521	GK13492	GI24602	GJ22665	GH18102
asp	CG6875	GD21126	GM26622	GE23511	GG11315	GF23045	GA26850	GL13623	GK14035	GI24024	GJ23654	GH18447
Axs	CG9703	GD15734	GM13377	GE15653	GG18235	GF21728	GA21976	GL12944	GK19948	GI15349	GJ19453	GH11928
c(2)M	CG4249	GD24050	GM18665	GE21442	GG25201	GF14579	GA18058	GL16321	GK23985	GI17074	GJ10618	GH10313
c(3)G	CG17604	GD20329	GM25754	GE26360	GG20377	-	GA26705	GL23692	GK10347	GI14995	GJ15351	GH12738
cav	CG6219	GD21077	GM26573	GE23460	GG11268	GF16116	GA27250;<br>GA26940	GL23417;<br>GL14051	GK11387;<br>GK24325	GI24179	GJ14215;<br>GJ17001	GH18668
CG7676	CG7676	GD19229	GM17867	GE25535;<br>GE14694	GG22880	-	GA26847	GL20209	-	-	-	-
Su(var)205	CG8409	GD22433	GM13138	GE11133	GG23468	GF15276	GA21056	GL19396	GK14980	GI15430	GJ19002	GH10251
Klp3A	CG8590	X_mrand<br>on170:2<br>-3067	GM18872	GE16938	GG12607	GF21944	GA21186	GL12941	GK10187	GI15286	GJ16941	GH24497
Ku70	CG5247	GD18751	GM23941	GE26092	GG18111	GF16859	GA18760	GL12608	GK11797	GI24114	GJ10937	GH18304
Ku80	CG18801	GD21940	GM14187	GE19388	GG24193	GF14974	GA15084	GL15436	GK24271	GI17244	GJ18003	GH13494
matrimony	CG18543	3L:7866<br>596-<br>7867240	scaffold<br>_0:70220<br>2-702846	3L:34916<br>82-<br>3492341	scaffold<br>_4784:56<br>25842-<br>5626495	scaffold<br>_13337:7<br>387752-<br>7388366	XR_group<br>6:128786<br>10-<br>12879221	scaffold<br>_33:9025<br>94-<br>903205	scf2_110<br>00000048<br>22:31016<br>20-<br>3102210;<br>GK15529-	scaffold<br>_6680:32<br>14531-<br>3215106	scaffold<br>_13049:4<br>823527-<br>4824117;<br>GJ19117	scaffold<br>_15110:1<br>0105685-<br>10106281

									RA			
mei-218	CG8923	GD15699	GM13338	GE15620	GG18203	GF22380	GA17258	GL20336	GK20079	GI15825	GJ18678	GH24636
mei-41	CG4252	GD17284	GM13435	GE17270	GG17962	GF12969	GA25526 and GA26566	GL14921	GK21910	GI18055	GJ22445	GH13804
mei-P22	CG14827	GD14002	GM14829	GE21603	GG14413	GF10572	GA13278	GL25236	GK19131	GI11952	GJ12177	GH15242
mei-S332	CG5303	GD11646	GM15884	GE12243	GG22163	GF13289	GA18794	GL11778	GK15937	GI20753; GI18821	GJ21848; GJ20489	GH20552; GH21335
mei-P26	CG12218	GD16934	GM13725	GE15827	GG18293	GF22313	GA11485	GL14648	GK25092	GI15721	GJ15250	GH24221
mei-W68	CG7753	GD11457	GM21963; GM13291	GE12053	GG21975	GF11715	GA24396	GL10528	GK15872	GI20732	GJ20469	GH20206
mei-9	CG3697	GD16302	GM12692	GE16862	GG18547	GF21474	GA17620	GL14183	GK17500	GI16132	GJ16410	GH24669
mre11	CG16928	GD23775	GM19007	GE18523	GG23718	GF14771	GA14221	GL19555	GK21058	GI20705; GI20694	GJ13289	GH13392
mus304	CG7347	GD14744	GM14964	GE19943	GG13649	GF23614	GA23899	GL22861	GK21090	GI16418	GJ16213	GH10057
ncd	CG7831	GD17657	GM12219	GE10426	GG12000	GF16326	GA20615	GL24007	GK11161	GI10718	GJ23134	GH20888
okr	CG3736	GD12001	GM18177	GE14966	GG24469	GF14336	GA17651	GL26701	GK15573	GI10760	GJ18280	GH10642
ord	CG3134	GD25069	GM15567	GE11589	GG20053	GF13305	GA16191	GL11285	scf2_110 00000045 12:21771 5-219356	GI21123	GJ20970	GH23052
polo	CG12306	GD14871	GM22270	GE19659	GG16099	GF10050	GA11545; GA25172; GA25958	GL25129; GL25881; GL19429	GK17233	GI11323	GJ11579	GH14380
rad50	CG6339	GD11670	GM15911	GE14184	GG22190	GF11892	GA19522	GL17546	GK20820	GI19621	GJ14987	GH20438
SMC1	CG6057	GD21061 and GD21062	GM26555	GE23444	GG11251	GF23004	GA19328	GL23399	GK11152	GI24164	GJ14197	GH18490
spn-A	CG7948	GD17366	GM12184	GE23416	GG11967	GF16205	GA20711	GL13479	GK13420	GI23388	GJ10382	GH18396

spn-B	CG3325	GD18959	GM24163	GE24233	GG16853	GF17509	GA17378	GL23505	GK10943	GI22807	GJ22809	GH17480
spn-D	CG31069	GD21329	GM10376	GE23723	GG11535	GF23286	GA15983	GL23908	GK13653	GI16495	GJ16038	GH17957
subito	CG12298	GD25444	GM19953	GE13965	GG21022	GF13227	GA24227	GL17361	GK16261	GI20445	GJ20115	GH19853
teflon	CG8961	GD25499	GM20010	GE14219	GG22222	GF13428	GA21437	GL10555	scf2_110 00000048 22:95806 3-960244	GI18435	GJ21520	GH22944
tefu	CG6535	GD20371	GM25794	GE26399	GG20781	GF11791	GA19668	GL12075 and GL12076	GK12780	GI11054	GJ14811	GH22366
