# Supplementary material for: Drosophila Genes That Affect Meiosis Duration Are among the Meiosis Related Genes That Are More Often Found Duplicated
Source: PLoS One. 2011 Mar 10;6(3):e17512. doi: 10.1371/journal.pone.0017512 (PMC3053365; doi:10.1371/journal.pone.0017512)
Supplement: Table S4 — Coding sequence size and intron number (in brackets) of 33 meiosis genes from 12 Drosophila species. (PDF) [file pone.0017512.s004.pdf]

Table S4. Coding sequence size and intron number (in brackets) of 33 meiosis genes from 12 *Drosophila* species. The + sign indicates that the gene is not annotated, but that can be detected using Blast.

	D. melanogaster	*D. simulans*	*D. sechellia*	*D. yakuba*	*D. erecta*	*D.ananassae*	*D. pseudoobscura*	*D. persimilis*	*D. willistoni*	*D. mojavensis*	*D. virilis*	*D. grimshawi*
ald	1893(3)	1686(2)	1902(3)	1899(3)	1899(3)	1902(3)	1947(3)	1947(3)	1923(3)	1911(4)	1875(3)	1941(4)
asp	5865(5)	3237(6)	5865(5)	5865(5)	5856(5)	5922(5)	5868(5)	5868(5)	5901(5)	5322(5)	5946(5)	5949(5)
Axs	1941(7)	1941(7)	1941(7)	1941(7)	1941(7)	1941(7)	1935(7)	1935(7)	1956(6)	1932(7)	1929(7)	1893(6)
c(2)M	1713(7)	1416(5)	1713(7)	1701(7)	1704(7)	1722(7)	1722(6)	1695(6)	534(1)	1689(7)	1512(6)	1443(7)
c(3)G	2235(7)	1914(5)	2235(7)	2232(7)	1395(4)	-	1995(3)	1536(5)	1455(6)	1587(5)	1812(6)	1395(5)
cav	1032(2)	1011(1)	1011(1)	960(1)	987(1)	942(0)	852(1); 561(1)	849(1); 561(1)	816(1); 768(1)	906(1)	906(1); 1173(1)	651(0)
CG7676	624(3)	585(2)	585(2)	585(2); 573(2)	600(2)	-	606(2)	606(2)	-	-	-	-
Su(var)205	621(3)	621(3)	621(3)	618(3)	618(3)	633(3)	618(3)	618(3)	618(3)	681(3)	642(3)	612(3)
Klp3A	3639(4)	+ 2934(2)	3657(4)	3672(4)	3645(4)	3744(4)	6894(10)	3645(4)	3597(4)	3474(4)	3612(5)	3408(4)
Ku70	1896(1)	1896(1)	1893(1)	1896(1)	1896(1)	1896(1)	1902(1)	1902(1)	1893(1)	1896(2)	1875(2)	1875(2)
Ku80	2100(4)	2100(4)	2100(4)	2100(4)	2067(4)	2100(4)	2079(3)	2079(3)	2073(3)	2103(4)	2106(4)	2136(4)
matrimony	654(0)	+ 645(0)	+ 645(0)	+ 660(0)	+ 654(0)	+ 615(0)	+ 612(0)	+ 612(0)	+ 591(0); 561(0)	+ 576(0)	+ 591(0); 567(0)	+ 597(0)
mei-218	3561(4)	711(2)	3639(4)	4653(10)	4098(9)	6033(8)	3009(4)	2133(2)	4494(8)	3273(4)	3291(5)	3141(5)

mei-41	7554(4)	7737(4)N	5835(2)	7560(4)	7551(4)	7548(4)	888(1);<br>4473(3)	7383(5)	7599(4)	7680(6)	7701(5)	7722(5)
mei-P22	996(0)	1005(0)	996(0)	993(0)	990(0)	972(0)	1428(0)	1428(0)	981(0)	669(0)	1455(0)	771(1)
mei-S332	1206(1)	1206(1)	1206(1)	1221(1)	1212(1)	1149(1)	1206(1)	1206(1)	1200(0)	540(0);<br>1260(1)	1308(1);<br>1305(1)	1086(1);<br>1095(1)
mei-P26	3570(5)	369(2)	3615(6)	3561(5)	3567(5)	3567(6)	3657(5)	3645(5)	3936(5)	3546(5)	3543(5)	3768(5)
mei-W68	996(1)	996(1)	996(1);<br>996(1)	996(1)	996(1)	993(1)	1002(1)	1002(1)	1041(1)	978(2)	1050(1)	1005(2)
mei-9	2886(5)	1809(2)	240(1)	2898(5)	2898(5)	2919(5)	2922(5)	2922(5)	2994(5)	2901(5)	2976(5)	2967(5)
mre11	1863(4)	1863(4)	1863(4)	1866(3)	1863(4)	1821(4)	1866(4)	1866(4)	1860(3)	1854(4)<br>1554(3)	1872(4)	1866(4)
mus304	2541(3)	2202(3)	2544(3)	2550(3)	2544(3)	2535(3)	2547(3)	2547(3)	2487(4)	2514(3)	2532(3)	2427(4)
ncd	2103(2)	2037(3)	2118(2)	2106(2)	2103(2)	2109(2)	2109(2)	2118(2)	2073(2)	2109(2)	2097(2)	2076(2)
okr	2355(5)	2352(5)	2154(6)	2319(4)	2355(5)	2376(5)	2349(5)	2349(5)	2355(5)	2352(5)	2361(5)	2361(5)
ord	1440(5)	1428(5)	1221(5)	1449(5)	1452(5)	1485(4)	1428(5)	1428(5)	+ 1299(4)	1425(5)	1413(5)	1407(5)
polo	1731(4)	1677(5)	1731(4)	1956(5)	1731(4)	1728(4)	1881(4);<br>1704(3);<br>1158(0)	1728(4);<br>1704(3);<br>1158(0)	1728(4)	1728(4)	1728(4)	1728(4)
rad50	3957(7)	3906(8)	3912(7)	3912(6)	3912(7)	3927(6)	3939(9)	3939(9)	3963(8)	3939(9)	3939(9)	3924(9)
SMC1	3117(10)	1290(3)<br>2403(8)	3585(11)	3717(10)	3717(10)	3711(10)	3717(10)	3708(11)	3714(10)	3723(10)	3723(10)	3723(10)
spn-A	1011(1)	1011(2)	1011(2)	1008(2)	1008(2)	1005(2)	1008(2)	1008(2)	1068(2)	1044(2)	1056(2)	1059(2)
spn-B	1026(3)	1026(3)	1026(3)	1026(3)	1026(3)	1038(2)	1047(3)	1047(3)	1050(2)	1038(3)	1050(3)	1050(3)
spn-D	813(5)	804(5)	813(5)	1221(7)	576(4)	705(5)	765(4)	576(4)	810(3)	546(3)	738(4)	741(4)
subito	1887(2)	1887(2)	1887(2)	1893(2)	1887(2)	1866(3)	1863(3)	1863(3)	1830(3)	1815(3)	1821(3)	1818(3)
teflon	1950(2)	1977(2)	1971(2)	1947(2)	1977(2)	1155(2)	1458(3)	2763(3)	+	1176(1)	1731(4)	2004(3)

tefu	8304(27)	8085(26)	8034(27)	8235(28)	8097(26)	7977(26)	8181(27)	4692(14)<br>2406(10)	8376(27)	6633(22)	8391(27)	8385(0)
